# Plasma-Catalytic CO_2_ Reforming of Toluene over Hydrotalcite-Derived NiFe/(Mg, Al)O_*x*_ Catalysts

**DOI:** 10.1021/jacsau.2c00603

**Published:** 2023-02-17

**Authors:** Lina Liu, Jing Dai, Sonali Das, Yaolin Wang, Han Yu, Shibo Xi, Zhikun Zhang, Xin Tu

**Affiliations:** † College of Environmental Science and Engineering, Ministry of Education Key Laboratory of Pollution Processes and Environmental Criteria, Nankai University, Tianjin 300350, China; ‡ Department of Chemical and Biomolecular Engineering, National University of Singapore, Singapore 117585, Singapore; § Department of Electrical Engineering and Electronics, University of Liverpool, Liverpool L69 3GJ, U.K.; ∥ Institute of Chemical and Engineering Sciences, A* STAR, 1 Pesek Road, Jurong Island, Singapore 627833, Singapore; ⊥ School of Energy and Environmental Engineering, Tianjin Key Laboratory of Clean Energy and Pollution Control, Hebei University of Technology, Tianjin 300401, China

**Keywords:** CO_2_ reforming of toluene, syngas, hydrotalcite-derived catalysts, plasma catalysis, in situ FTIR

## Abstract

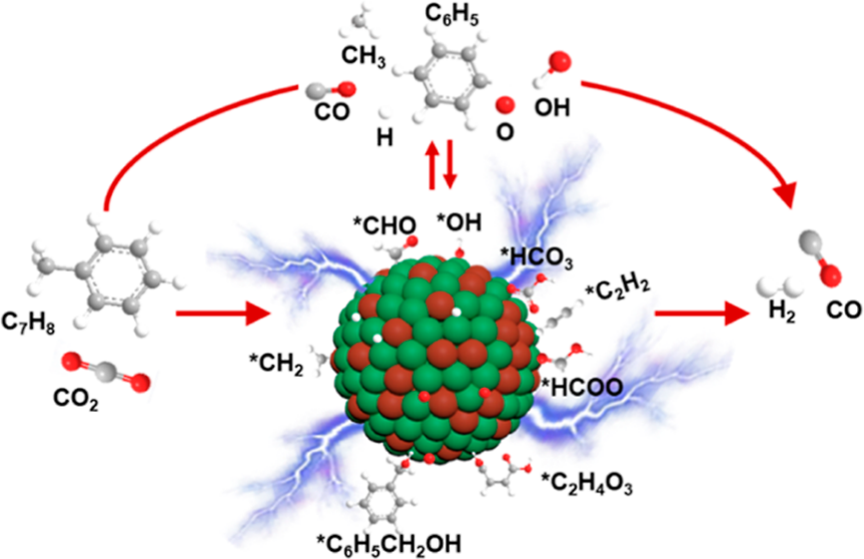

The removal of tar and CO_2_ in syngas from biomass gasification is crucial for the upgrading and utilization of syngas. CO_2_ reforming of tar (CRT) is a potential solution which simultaneously converts the undesirable tar and CO_2_ to syngas. In this study, a hybrid dielectric barrier discharge (DBD) plasma-catalytic system was developed for the CO_2_ reforming of toluene, a model tar compound, at a low temperature (∼200 °C) and ambient pressure. Periclase-phase (Mg, Al)O_*x*_ nanosheet-supported NiFe alloy catalysts with various Ni/Fe ratios were synthesized from ultrathin Ni–Fe–Mg–Al hydrotalcite precursors and employed in the plasma-catalytic CRT reaction. The result demonstrated that the plasma-catalytic system is promising in promoting the low-temperature CRT reaction by generating synergy between DBD plasma and the catalyst. Among the various catalysts, Ni4Fe1-R exhibited superior activity and stability because of its highest specific surface area, which not only provided sufficient active sites for the adsorption of reactants and intermediates but also enhanced the electric field in the plasma. Furthermore, the stronger lattice distortion of Ni4Fe1-R provided more isolated O^2–^ for CO_2_ adsorption, and having the most intensive interaction between Ni and Fe in Ni4Fe1-R restrained the catalyst deactivation induced by the segregation of Fe from the alloy to form FeO_*x*_. Finally, in situ Fourier transform infrared spectroscopy combined with comprehensive catalyst characterization was used to elucidate the reaction mechanism of the plasma-catalytic CRT reaction and gain new insights into the plasma-catalyst interfacial effect.

## Introduction

Thermochemical conversion of renewable biomass via a gasification process is a promising and emerging carbon-neutral technology to produce synthesis gas (syngas) for power and synthetic fuel applications. However, the produced syngas contains not only H_2_ and CO but also CO_2_, CH_4_, and tar.^1^ In spite of continuous efforts in recent decades, the presence of undesirable tar in syngas (1–100 g/m^3^) remains the most challenging technological constraint for upscaling biomass gasification.^2^ As a complex mixture of condensable aromatic hydrocarbons, tar will cause several problems upon cooling and condensation, such as the reduction of overall energy efficiency and the clogging and fouling of downstream equipment and pipelines.^3^

Tar can be eliminated by various techniques, such as mechanical separation, thermal cracking, and thermal-catalytic reforming.^4^ Among them, thermal-catalytic tar reforming is the most extensively investigated, this process converts tar into valuable syngas in the presence of a reforming medium (e.g., H_2_O and CO_2_) and a catalyst. Considering the complexity of real biomass tar, model tar compounds, including benzene, toluene, and naphthalene, are generally employed for catalyst design and mechanism investigation.^5−7^ Nevertheless, both steam and CO_2_ reforming reactions (eqs 1 and 2) are endothermic and highly energy-intensive processes, which generally require high temperatures of 600–800 °C even with the aid of a catalyst, resulting in lower energy efficiency. In addition, there is also rapid catalyst deactivation caused by coke deposition and metal sintering, as well as high capital and operation costs. Therefore, it is critical to develop a low-temperature and energy-efficient process for tar reforming.
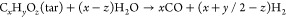
1
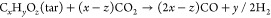
2

Nonthermal plasma (NTP) has gained increasing attention as an emerging technology for gas conversion. NTP is generated by electric power; the applied electrical energy mainly heats the electrons, rather than the gas as a whole. Thus, a significant thermal nonequilibrium can be achieved between the highly energetic electrons (typically up to several tens of thousands of Kelvin) and the gas molecules (which may even remain at room temperature). Subsequently, these energetic electrons will induce the excitation, ionization, and dissociation of the gas molecules by collision, which enable thermodynamically unfavorable or energy-intensive reactions to occur even at ambient temperature and pressure.^8^ NTP has been investigated for various environmental and energy-related processes, such as volatile organic compound removal, CO_2_ hydrogenation, and reforming reactions. However, it typically suffers from poor selectivity of target products because of the concurrence of multiple side reactions.^9^ The integration of NTP with a suitable catalyst in a hybrid system is a promising strategy for generating synergy, that is, low temperature and fast activation of reactants by NTP and selective formation of desired products using the catalyst.^10^ Previous research has demonstrated that toluene conversion (91.7%) in the dielectric barrier discharge (DBD) plasma-catalytic reforming of toluene over a Ni/γ-Al_2_O_3_ catalyst was substantially higher than the sum of the toluene conversion (56.0%) achieved by the individual plasma and catalytic systems.^11^ Despite the obvious observed synergistic effect of the plasma and catalyst, the essence and mechanism of plasma-catalysis interactions during tar reforming has rarely been demonstrated due to the lack of available in situ characterization techniques. Furthermore, it is well accepted that developing efficient catalysts suitable for plasma-enabled reactions is critical to enhancing the plasma-catalysis synergy. Currently, however, the knowledge in designing efficient catalysts for this process remains unclear.^12^

Supported Ni catalysts are the most promising catalysts in thermal-catalytic tar reforming due to their economic viability and high reactivity in C–C and C–H bond activation. However, they are restricted by the rapid catalyst deactivation caused by coke deposition and metal sintering at high temperatures.^5^ Strategies such as alloying with a secondary metal, adding a promoter (rare earth oxides, alkali and alkaline earth metals), modifying the supports, and optimizing the preparation methods have been widely investigated in order to enhance the thermal stability of Ni-based catalysts.^13^ However, the preparation and modification of catalysts for plasma reactions should not completely follow the same principle as those for thermal reactions since a catalyst might behave differently in plasma and thermal reactions.^14^ For instance, our previous study demonstrated that the K- and Ce-promoted Ni/Al_2_O_3_ catalysts enhanced the conversion of CO_2_ compared to Ni/Al_2_O_3_, while the use of Mg-promoted catalysts had a negative impact on CO_2_ conversion in plasma-catalytic biogas reforming. However, K-, Mg-, and Ce-promoted catalysts showed antipodal activities in thermal-catalytic biogas reforming, where only the Mg-promoted Ni catalyst promoted CO_2_ conversion. Such a difference can be attributed to the temperature-dependent character of the promoters in reforming reactions since the Mg-promoted catalyst exhibited decreased basic sites and weaker CO_2_ affinity at low temperatures. Furthermore, we also found that the metal size and dispersion of catalysts played a high priority role in accelerating the conversion of CH_4_ and CO_2_ in DBD plasma.^9,15,16^ Therefore, more attention should be paid to the low-temperature characteristics of catalysts in plasma reactions. Layered double hydroxides (LDHs), also known as hydrotalcites, with a general formula of [M_1–*x*_^2+^M_*x*_^3+^(OH)_2_]^*x*+^[A_*x*/*n*_]^*n*−^·*m*H_2_O, are a family of two-dimensional nanostructured anionic clays comprising of brucite-like layers. Upon high-temperature calcination, they are easily transformed into mixtures of metal oxides with large surface areas, high metal dispersion, tunable metal loading, and excellent basicity.^17,18^ These properties make them attractive as catalyst precursors in tar reforming reactions. However, the performance of LDH-derived catalysts has rarely been reported in plasma-catalyzed tar reforming reactions. Furthermore, most previous studies preferred to use steam as a reforming agent, rather than CO_2_. However, the role of CO_2_ as a reforming agent should not be neglected especially as it is a typical component in syngas with a content of 15–25 vol %.^19^ The CO_2_ reforming of tar might be a more promising route due to increasing the available carbon in biomass and circumventing additional CO_2_ separation processes before syngas utilization.^20^

Herein, the CO_2_ reforming of toluene (CRT) as a model tar surrogate was investigated in a DBD plasma-catalytic system (Figure S1), and a LDH-derived Ni-based catalyst supported on a periclase-phase (Mg, Al)O_*x*_ nanosheet was synthesized and employed (Table S1). The partial substitution of Ni by Fe was performed to prepare bimetallic catalysts with various Ni/Fe ratios (1:1, 2:1, 3:1, 4:1, and 1:0). In addition, the effect of CO_2_ content on the performance of CRT was evaluated. Most importantly, in situ Fourier transform infrared (FTIR) spectroscopy characterization of the plasma-catalytic CRT was conducted for the first time to provide new insights into the synergistic reaction mechanism of DBD plasma and catalysts.

## Results and Discussion

### Crystalline Phase and Element Compositions

**Figure 1:**
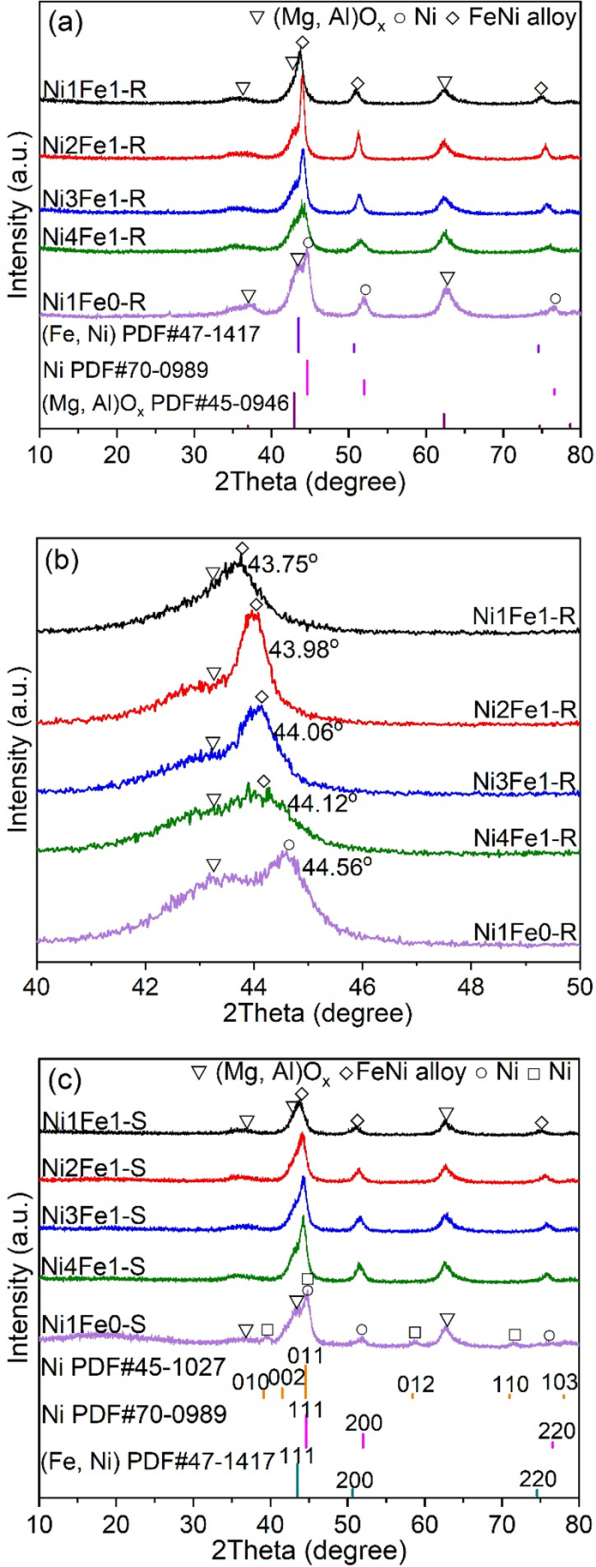
XRD patterns of (a,b) reduced and (c) spent LDH-derived NiFe/(Mg, Al)O_*x*_ catalysts.

The crystalline phase compositions of the as-synthesized LDH precursors and fresh calcined and reduced LDH-derived catalysts are depicted in Figures 1 and S2. Figure S2a illustrates that all Ni–Fe–Mg–Al LDH precursors presented characteristic X-ray diffraction (XRD) patterns of pure hydrotalcite crystal structures. When examining the XRD patterns, the peak at 2θ of ∼11.5°, indexed to the (003) plane of Ni–Fe–Mg–Al LDHs (Figure S2b), underwent a gradual shift in position from 11.29° to higher angles of 11.40–11.63° after Fe^3+^ doping with increases in the Fe/Ni molar ratio from 0 to 1, suggesting a variety in interlayer spacing within the LDH precursors. The *d*-spacing of (003) planes (i.e., the interlayer spacing) determined from the XRD patterns and Bragg’s law (Table S2) decreased from 0.7799 to 0.7615 nm with the increase of Fe loading, confirming the lattice shrinkage induced by the substitution of Ni^2+^ (0.69 Å) with the smaller Fe^3+^ (0.65 Å).^21^

Upon calcination at 600 °C, the characteristic peaks of LDHs disappeared, while periclase-like phases of the Mg(Ni, Fe, Al)O solid solution emerged (Figure S2c).^22^ For the XRD pattern of Ni1Fe0-F without any Fe addition, the periclase-like MgNiO_2_ solid solution was recognized as the major crystalline phase, as well as MgAl_2_O_4_ spinel (PDF#33-0853). With the addition of Fe, a newly formed phase of MgFe_2_O_4_ spinel could be observed (PDF#36-0398).^23^ The actual element compositions and Ni/Fe ratios of the calcined catalysts determined by inductively coupled plasma optical emission spectroscopy (ICP-OES) were consistent with the nominal values (Table S3), suggesting the reliability of the preparation method. Despite varying the Ni/Fe ratios, the total mass ratio of active Ni and Fe was maintained at a similar value of 19.3–20.1%.

**Scheme 1:**
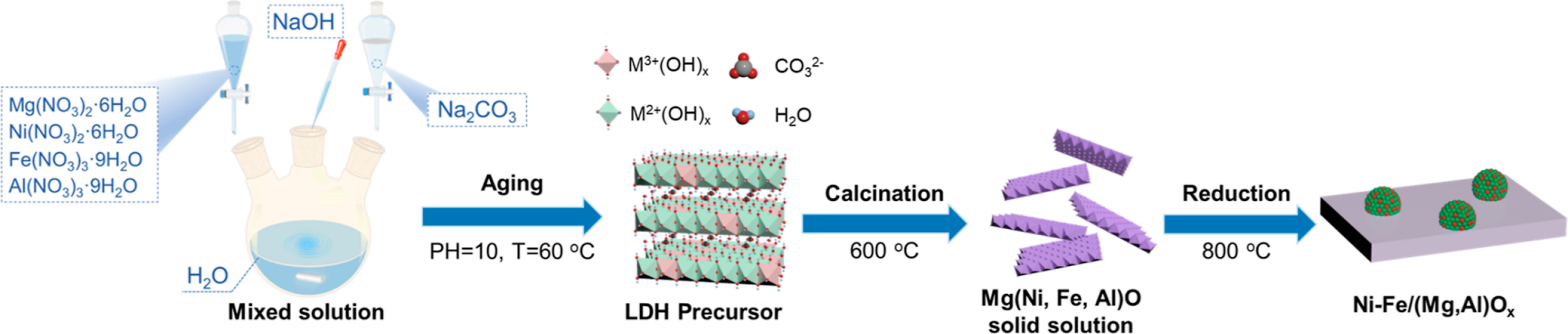
Schematic Illustration for the Preparation of LDH-Derived NiFe Alloys Anchored on a Periclase-Phase (Mg, Al)O_*x*_ Nanosheet

Figure 1a shows the XRD patterns of the reduced catalysts, where the periclase-like structure of (Mg, Al)O_*x*_ which appeared at 2θ of 36.9, 42.9, and 62.3° (PDF#45-0946) was detected as the catalyst support.^24^ No peaks related to Ni- and Fe-containing oxides were found, confirming that the active metals (Ni and Fe) were completely exsolved and reduced from the solid solution after the reduction. In addition to (Mg, Al)O_*x*_, diffraction peaks could be observed at 2θ of 44.6, 52.0, and 76.6° in Ni1Fe0-R, corresponding to the (111), (200), and (220) planes of metallic Ni with a face-centered cubic (fcc) structure (PDF#70-0989).^16^ These peaks were shifted to lower angles in the XRD patterns of the Fe-containing catalysts, indicating the formation of NiFe alloys (PDF#47-1417).^25^ The result confirmed the presence of an intimate interaction between Fe and Ni.^9^ The increase in Fe loading shifted the (111) reflections of metallic Ni(Fe) from 44.56 to 43.75°, as indicated by the magnified XRD patterns in Figure 1b. Furthermore, the compositions of alloys in the reduced catalysts were also determined using the *d*(2 0 0) spacing value of NiFe alloys in Figure 1a and Vegard’s law, as shown in Table 1. The calculated Ni/Fe ratios (1.14–3.96) in the alloys were close to the measured values (1.1–3.7) in Table S3, indicating that Ni and Fe could be reduced simultaneously, forming an alloy.^6^ The higher Ni/Fe ratios in alloys compared to bulk catalysts indicate that Ni was reduced more than Fe. Additionally, NiFe alloys (especially in the Ni4Fe1-R catalyst) presented broader diffraction peaks with lower intensities than bare Ni, which may be attributed to the distortion of the lattice and the underlying peak of the amorphous surface.^26^ This phenomenon implies a higher dispersion and a smaller particle size of NiFe alloys. Similar findings have been reported by Li et al. where the diffraction peaks of Ni became broader and gradually shifted to lower angles with an increasing Cu/Ni ratio, suggesting the formation of small Ni–Cu alloy nanoparticles (NPs).^7^ The average crystalline sizes of Ni and NiFe alloys in the catalysts were calculated from the full width at half-maximum of a diffraction peak originating from the fcc (200) facet using the Scherrer equation. The result confirmed that Ni1Fe1-R and Ni4Fe1-R exhibited the smallest crystalline sizes of 9.2 and 9.5 nm, respectively, while Ni1Fe0-R showed the largest size of 12.6 nm (Table 1). However, it is noteworthy that this value might be underestimated for alloys in the catalysts because of the heterogeneity of alloy composition.^6^ Based on the above analysis, the structural evolution of the Ni–Fe–Mg–Al LDH precursor to periclase-like mixed oxides by calcination at 600 °C and then to NiFe alloy NPs anchored on periclase-phase (Mg, Al)O_*x*_ nanosheets by H_2_ reduction at 800 °C is illustrated in Scheme 1.

**Table 1:** Textural Properties of the Fresh Calcined and Reduced Catalysts

	*S*_BET_^a^ (m^2^/g)		*V*_p_^a^ (cm^3^/g)		*D*_p_^a^ (nm)								Ni/Fe molar ratio in alloy^h^	
catalyst	fresh	reduced	fresh	reduced	fresh	reduced	crystalline size^b^ (nm)	particle size^c^ (nm)	H_2_ consumption^d^ (mmol/g)	reduction degree^e^ (%)	basicity^f^ (mmol/g)	surface Ni/Fe atomic ratio^g^	reduced	spent
Ni1Fe1	207.1	153.7	0.75	0.82	14.2	20.5	9.2	10.28	4.23	87.8	0.182	0.38	1.14	1.76
Ni2Fe1	217.3	162.3	0.72	0.80	13.2	18.1	10.6	10.75	4.08	91.0	0.174	0.76	2.11	2.44
Ni3Fe1	219.4	160.0	0.61	0.86	10.4	20.6	10.2	10.80	3.89	90.0	0.168	0.90	3.04	3.62
Ni4Fe1	211.3	166.1	0.63	0.71	11.2	16.7	9.5	10.69	3.69	87.4	0.373	1.16	3.96	4.13
Ni1Fe0	181.4	143.6	0.44	0.48	9.2	12.7	12.6	16.90	3.34	87.2	0.186			

a Calculated from N_2_ isotherms at 77 K.

b Calculated from XRD patterns using the Debye–Scherrer equation.

c Determined by the FETEM images.

d H_2_ consumption below 800 °C, determined by the H_2_-TPR profiles in Figure 2a.

e Determined by the ratio of actual H_2_ consumption to stoichiometric H_2_ consumption. The stoichiometry of the Ni and Fe reductions is Ni^2+^ + H_2_ → Ni^0^ + 2H^+^ and Fe^3+^ + 3/2H_2_ → Fe^0^ + 3H^+^, respectively.

f Determined by the CO_2_-TPD profiles in Figure 2b.

g Atomic ratios of Ni and Fe on the surface of the reduced catalyst determined by XPS.

h Atomic ratios of Ni and Fe in alloy particles determined by the *d*(2 0 0) spacing derived from XRD and Vegard’s law (Figure 1a).

### Texture Properties

Figure S3 displays the N_2_ adsorption–desorption isotherms and pore diameter distributions of the fresh calcined and reduced LDH-derived catalysts, respectively. Both calcined and reduced catalysts presented type IV isotherms with H3-type hysteresis loops, indicating the presence of mesoporous structures with slit-shaped pores generated by aggregation of mixed-oxide platelets.^27^ However, the hysteresis loops of reduced catalysts were remarkably narrower compared to those of calcined catalysts. The results showed that the mesoporous structures were partially destroyed during the reduction, resulting in larger pores forming between compacted aggregates,^28^ as confirmed by the field emission scanning electron microscopy (FESEM) images in Figures 3a,b and S4.

**Figure 2:**
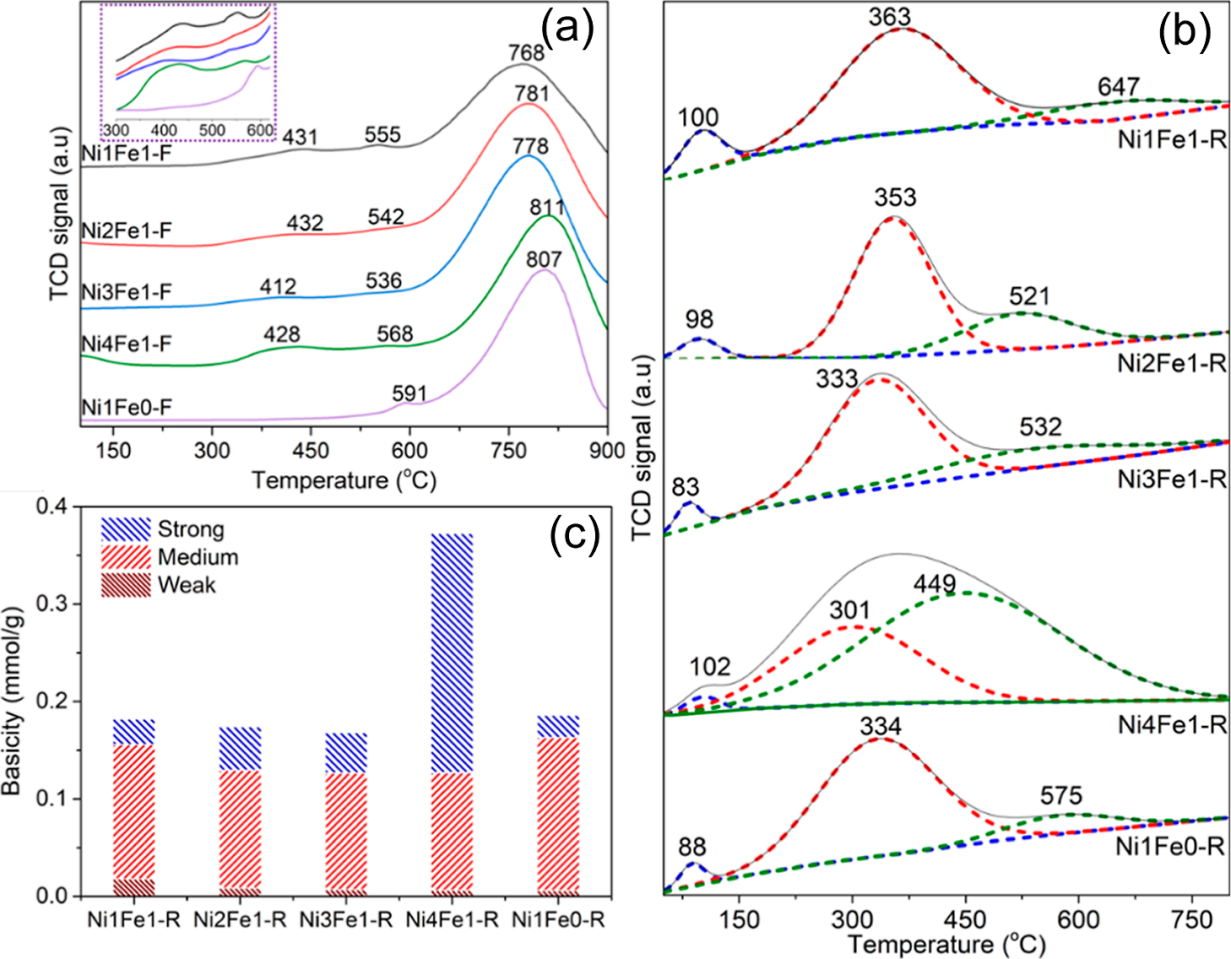
(a) H_2_-TPR, (b) CO_2_-TPD, and (c) basicity of the Ni–Fe/(Mg, Al)O_*x*_ catalysts.

**Figure 3:**
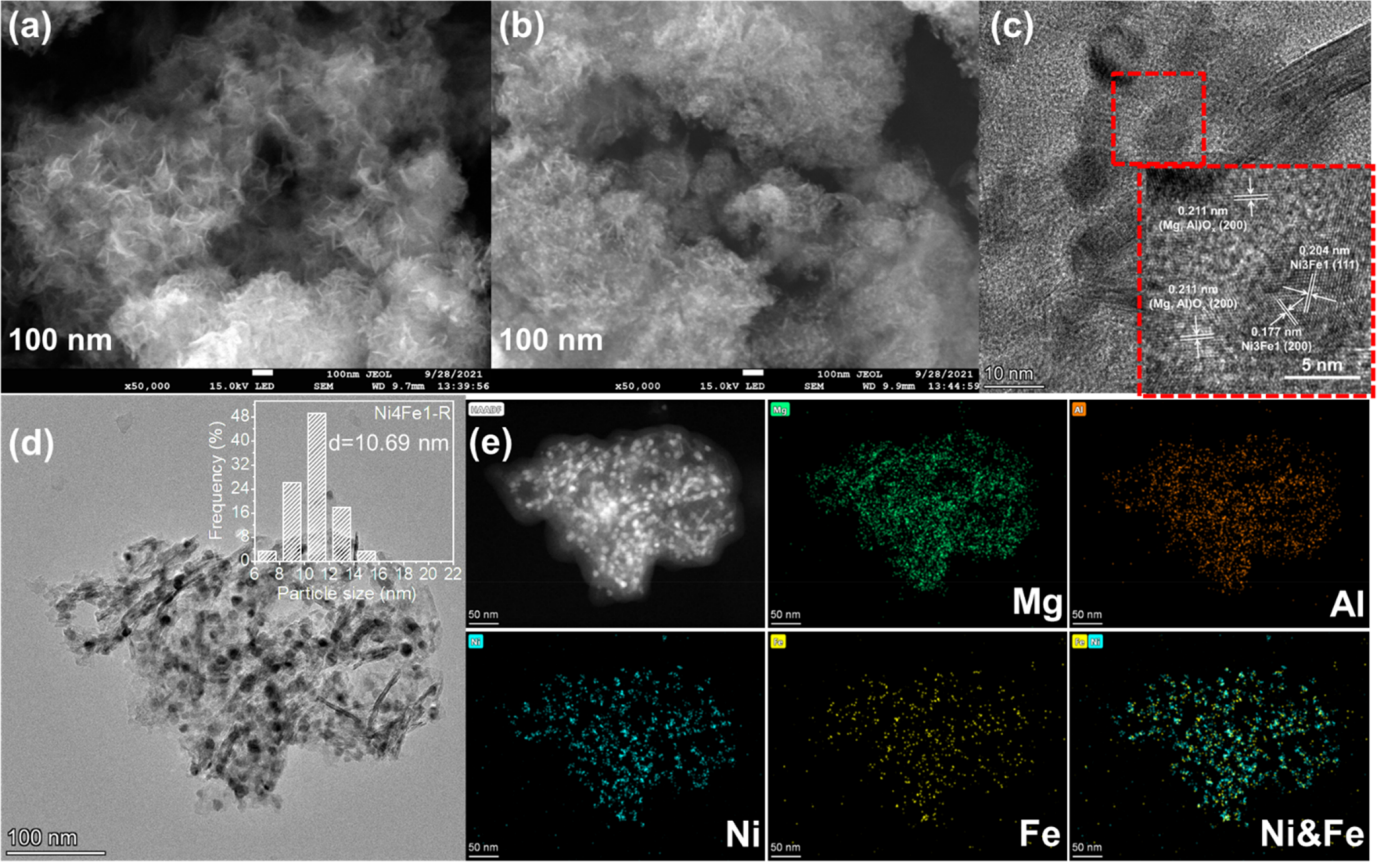
FESEM images of (a) calcined and (b) reduced Ni4Fe1/(Mg, Al)O_*x*_ catalysts; (c) magnified FETEM images and lattice fringes, (d) nanoparticle size distribution, and (e) EDX-mapping images of reduced Ni4Fe1/(Mg, Al)O_*x*_ catalyst.

Table 1 summarizes the typical texture properties of the catalysts extracted from the isotherms, including the specific surface area, total pore volumes, and average pore diameters. The calcined catalysts had relatively high specific surface areas ranging from 181.4 to 219.4 m^2^/g. Among the various catalysts, Ni1Fe0-F possessed the smallest surface area of 181.4 m^2^/g and the smallest pore volume of 0.44 cm^3^/g. The addition of Fe contributed to the significant increase in the surface area and pore volume. It should be noted that the specific surface area decreased while the mesopore volume increased after catalyst reduction. This phenomenon could be explained by the fact that, as previously stated, the extraction of metallic Ni and NiFe alloys from the mixed oxide platelets resulted in the collapse and merger of partial mesopores into larger pores. Ni4Fe1-R exhibited the highest specific surface area of 166.1 m^2^/g, which may favor the enhancement of catalytic performance by improving the dispersion of metal particles.

### Reducibility and Basicity

The reducibility of the LDH-derived catalysts determined by the H_2_-temperature-programmed reduction (H_2_-TPR) profiles is shown in Figure 2a and their corresponding H_2_ uptakes and reduction degrees are listed in Table 1. The Ni1Fe0-F catalyst exhibited two reduction peaks at 591 and 807 °C, which can be assigned to the reduction of surface NiO and stable NiO species interacting firmly with (Mg, Al)O_*x*_ periclase supports (MgNiO_2_ solid solution, as shown in Figure S2c), respectively.^29,30^ The incorporation of Fe resulted in the appearance of an additional peak at 412–432 °C, which is related to the reduction of surface Fe^3+^ to Fe^2+^, as shown in the enlarged H_2_-TPR profiles at 300–600 °C. For the Fe-incorporated catalysts, the major reduction peaks centered at 768–811 °C can be attributed to the coreduction of surface Fe^2+^ and bulk Fe^3+^ in the MgFe_2_O_4_ spinel phase to Fe^0^, as well as bulk Ni^2+^ in MgNiO_2_ solid solution to Ni^0^.^31^ The temperature of the reduction peaks decreased while their areas increased with the increase in Fe loadings, confirming the enhanced reducibility of the catalysts induced by the strong interaction between Ni and Fe due to the formation of the NiFe alloy, as confirmed in Figure 1a.^32^ Among all catalysts, Ni4Fe1-F presented the highest reduction temperature of the major peak at 811 °C. This phenomenon can be explained by the strongest metal–support interaction, which facilitated the formation of the NiFe alloy with high dispersion and finite size.^33^ In addition, the enhanced metal–support interaction is generally responsible for the superior catalytic performance and coke resistance.^34^

It has previously been reported that LDH-derived catalysts generally have an abundance of basic sites, which are beneficial to the adsorption of acidic reactants (e.g., CO_2_).^29^ The adsorbed CO_2_ molecules could provide a large number of active surface oxygen species, which was conducive to the gasification of carbon intermediates and hence accelerated the elimination of the generated surface carbon species, contributing to the superior coke resistance of catalysts.^35^ Therefore, CO_2_-temperature-programmed desorption (CO_2_-TPD) was employed to examine the basicity of the Ni–Fe/(Mg, Al)O_*x*_ catalysts, as illustrated in Figure 2b. The CO_2_-TPD profiles could be deconvoluted into three Gaussian peaks, corresponding to the weak, moderate, and strong basic sites, respectively. The weak Bronsted basic site centered at 83–102 °C is assigned to surface hydroxyl (OH^–^) groups, while the moderate Lewis basic site at 301–363 °C is associated with O^2–^ coordinated with Mg^2+^ cations. The strong Lewis basic site at 449–647 °C is related to the coordination-unsaturated O^2–^ species bound to Mg^2+^ cations.^36^ The distribution and number of basic sites, calculated by the integration of desorption peaks, are displayed in Figure 2c and Table 1. Among the various basic site categories, the moderate basic sites were the most dominant for almost all of the catalysts, which is consistent with the observations by Ren and Liu.^29^ However, Ni4Fe1-R presented more strong basic sites, which should be interpreted as a result of its higher lattice distortion, as confirmed by the XRD result in Figure 1b, contributing to the formation of more isolated O^2–^. In addition, its smaller crystallite size and higher surface area also contributed to its higher basicity.^17^

### Morphologies

The overall morphologies of the as-synthesized LDH precursors and fresh calcined and reduced Ni–Fe/(Mg, Al)O_*x*_ catalysts were measured using FESEM and field emission transmission electron microscopy (FETEM) with energy-dispersive X-ray spectroscopy (EDX) elemental mapping. The Ni4Fe1–Mg–Al LDH precursor was composed of an ultrathin sheetlike nanostructure with a smooth and flexible surface (Figure S4a). The thickness of the nanosheet was estimated to be 1.2–1.6 nm by atomic force microscopy (AFM), as shown in Figure S4e,f, which was much smaller than the 0.03–0.05 μm reported in the previous study.^37^ These ultrathin LDH precursors favor the improvement of catalyst morphology and surface area and promote the surface exposure of more NiFe alloy NPs with high homogeneity and dispersion, which can facilitate the reaction process.^38^ It is interesting to note that the extended sheetlike structure was fragmented into many smaller nanosheets with a loose and interlaced structure by calcination (Figure 3a). There was no obvious agglomeration or sintering of adjacent nanosheets. After H_2_ reduction, the overall nanosheet morphology with an open porous structure remained relatively intact, whereas numerous NPs dispersed on the nanosheet appeared because of the extraction of NiFe alloys from the mixed-oxide arrays (Figure 3b). The abundant spacings formed between thin surface nanosheets could facilitate the transformation of reactants and products in heterogeneous reactions.^39^ The FETEM images of the NixFe1-R catalysts confirmed the formation of well-dispersed NPs that were supported or embedded on the nanosheet support (Figures S5a–d and 3d). The magnified FETEM image of the Ni4Fe1-R catalyst displays three distinct lattice fringes with interplanar spacings of 0.177, 0.204, and 0.211 nm (Figure 3c). The former two correspond to the (200) and (111) planes of the fcc bimetallic NiFe alloy phase, while the latter can be assigned to the (200) plane of the periclase-phase (Mg, Al)O_*x*_ nanosheet support.^40^ The size distribution of the NiFe alloy NPs achieved from the TEM images shows a slight change from 10.28 to 10.80 nm when increasing the Ni/Fe molar ratio, which is consistent with the crystalline size obtained from the XRD results. Furthermore, the EDX mapping result in Figure 3e clearly shows the homogeneous distributions of Ni, Fe, Mg, and Al. Specifically, Ni and Fe elements over NPs presented very similar distributions.

However, the Ni–Mg–Al precursor without any Fe addition exhibited a relatively dense and inhomogeneous structure, which possessed some irregular aggregates with a coarser surface, in addition to the disconnected nanosheets (Figure S4b). After calcination and reduction, the porous nanosheet appearance was almost completely destroyed and metallic Ni in the interior of the support was exposed.^41^ Sintering was clearly visible in both metal NPs and support (Figure S4d). Meanwhile, the FETEM image of the Ni1Fe0-R catalyst in Figure S5d reveals a much larger NP size of ∼16.9 nm. Its well-resolved lattice fringe was measured to be 0.203 and 0.211 nm, which could be indexed to the (111) plane of metallic Ni and the (200) plane of (Mg, Al)O_*x*_, respectively (Figure S5).^9^ The clear difference in the morphology caused by the absence of Fe is consistent with the texture properties determined by N_2_ adsorption–desorption isotherms.

### Surface Chemical Properties

**Figure 4:**
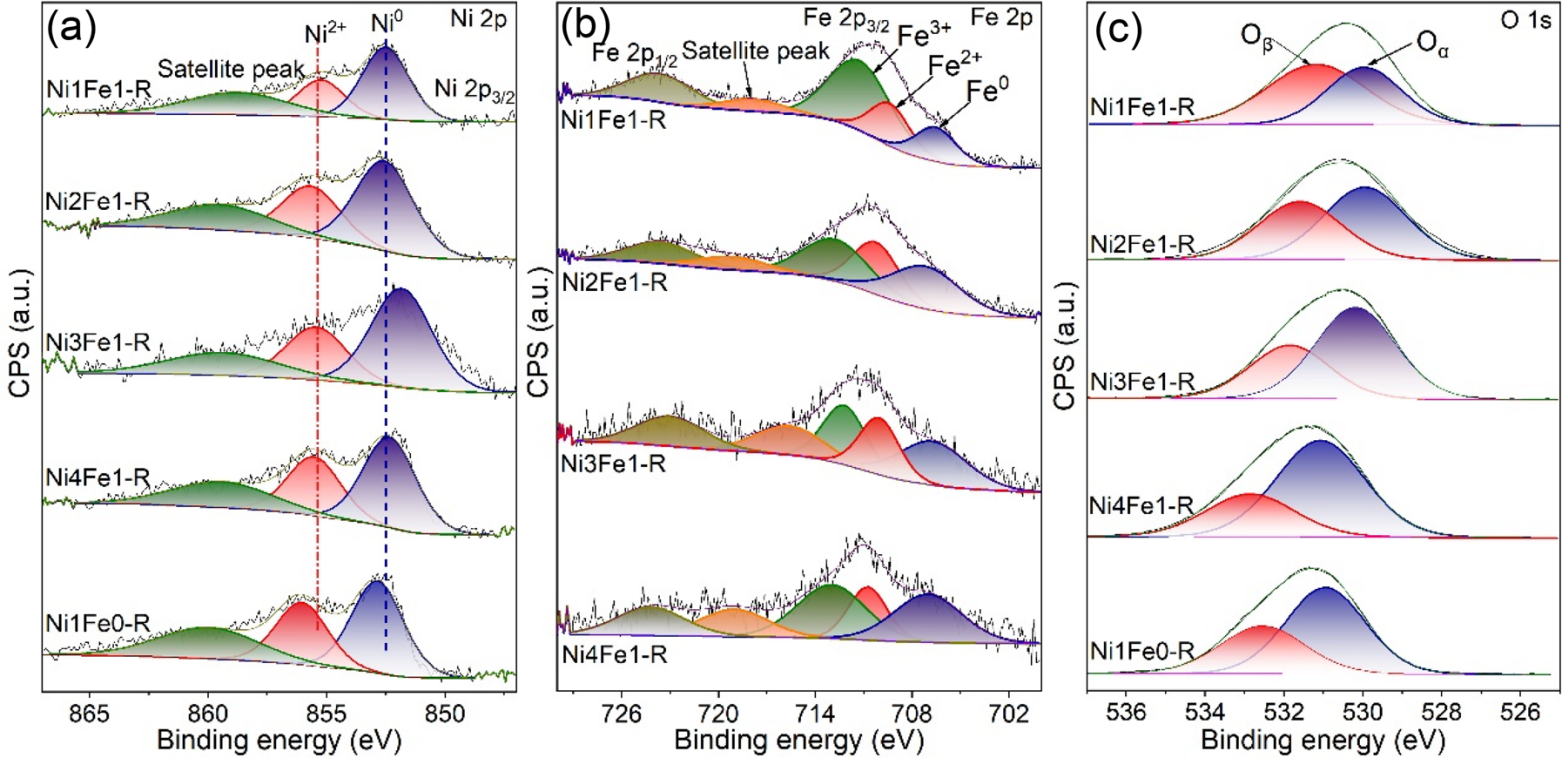
(a) Ni 2p, (b) Fe 2p, and (c) O 1s XPS spectra of reduced Ni–Fe/(Mg, Al)O_*x*_ catalysts.

X-ray photoelectron spectroscopy (XPS) was used to investigate the electronic states and chemical compositions of surface elements in reduced Ni–Fe/(Mg, Al)O_*x*_ catalysts (Figure 4 and Table S4). As shown in Figure 4a, the high-resolution Ni 2p_3/2_ XPS spectra of all reduced catalysts could be deconvoluted into three peaks, that is, Ni^0^ at 852.0–852.8 eV, Ni^2+^ at 855.2–856.0 eV, and the accompanying shake-up satellite peaks at ∼860 eV.^41^ Metallic Ni^0^ was identified as the dominant Ni species with contents of 59.6–66.3%. Notably, the binding energy of Ni^0^ was shifted to lower values, and the intensity of Ni^0^ increased with the incorporation of Fe (Table S4). This phenomenon confirms the presence of a strong electronic interaction between Ni and Fe, implying the formation of NiFe alloys in reduced Ni–Fe/(Mg, Al)O_*x*_ catalysts.^42^ The presence of Fe may alter the Ni electronic environment, resulting in an electronically richer state of Ni compared to the Ni/(Mg, Al)O_*x*_ catalyst.^38^ It has been previously reported that the enhanced electron density of Ni could help activate reactants and balance the adsorption of C* and O* species over the catalyst, contributing to the higher catalytic performance of Ni–Fe/(Mg, Al)O_*x*_ catalysts.^43^

Consistently, the binding energy of Fe^0^ observed from Fe 2p XPS spectra of the Ni–Fe/(Mg, Al)O_*x*_ catalysts shifted to higher values compared to the standard value of 706.7 eV (Figure 4b and Table S4). In addition to Fe^0^, Fe^2+^ and Fe^3+^ could be found at ∼710 and ∼712 eV, respectively.^44^ Among various catalysts, the Ni4Fe1-R catalyst had the largest Fe^0^ content at the highest binding energy of 707.3 eV, suggesting the strongest interaction between Ni and Fe, which was more difficult to oxidize. It should be noted that the calculated atomic ratios of Ni and Fe (0.38–1.16) on the surface of reduced catalysts from XPS data were much lower than those in the bulk phase. This could be attributed to the fact that the ratios of Ni and Fe on the catalyst surface were more affected by the oxide phases because of their high surface concentrations. The lower Ni/Fe ratio on the surface suggests that Ni was more reduced to the metallic state, whereas Fe tended to be easily oxidized on the surface.

The deconvolution of O 1s core level spectra yields two components, as illustrated in Figure 4c. Peaks at ∼532 eV correspond to the oxygen atoms bound to hydroxyl species, while peaks at ∼530 eV are associated with the defect sites in low oxygen coordination.^41^ Ni4Fe1-R has the highest ratio (69.2%) of defect sites, determined by the relative peak area, indicating the abundant oxygen vacancies induced by the doping of a small amount of Fe. It could be explained by the high and flexible coordination number of iron ions that altered the topology of the layer and hence caused the lattice distortion and laminar imperfections.^38^ However, excessive Fe doping decreased the oxygen vacancies, as listed in Table S4. It can be attributed to two reasons: (1) the presence of excessive Fe will weaken the asymmetry and lattice distortion since the limited space composed of six NiO_6_ octahedra was unable to accommodate too many distorted FeO_6_ octahedra, forcing the dissolution of Fe cations to release the lattice distortion, as revealed by the XRD and CO_2_-TPD results^45^ and (2) the presence of excessive Fe can promote the adsorption of oxygen-containing species on the catalyst surface due to its oxygen affinity.^46^

### XAS Analysis

**Figure 5:**
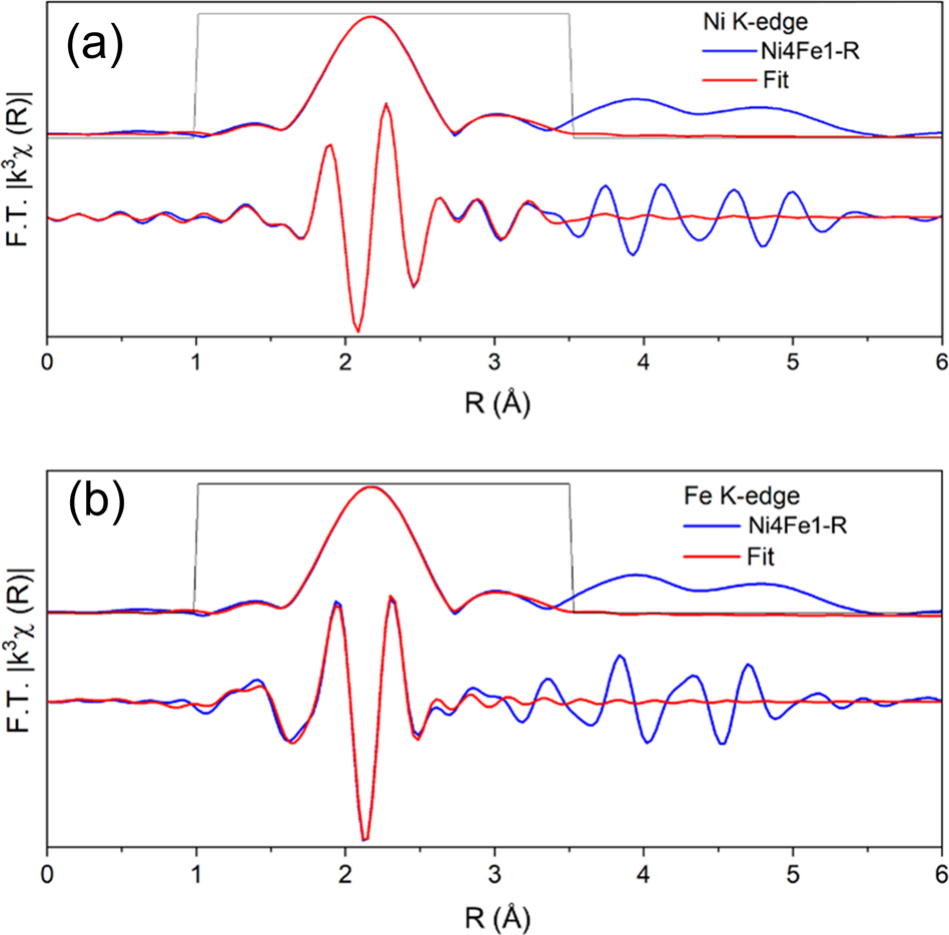
Magnitude and imaginary part of the *k*^3^-weighted Fourier transform of (a) Ni K-edge EXAFS spectrum and fit of reduced Ni4Fe1 (Fourier transform was taken over *k* = 3.3 to 11, and the fitting was done from *R* = 1 to 3.5. The amplitude reduction factor was calculated to be 0.79 from fitting the EXAFS spectrum for the Ni foil) and (b) Fe K-edge EXAFS spectrum and fit of reduced Ni4Fe1 (Fourier transform was taken over *k* = 3 to 12, and the fitting was done from *R* = 1 to 2.9. The amplitude reduction factor was calculated to be 0.7 from fitting the EXAFS spectrum for the Fe foil).

X-ray absorption spectroscopy (XAS) at the Ni and Fe K edges was conducted to gain further understanding of the state of Ni and Fe in Ni4Fe1/(Mg, Al)O_*x*_ after reduction. X-ray absorption near edge spectroscopy (XANES) spectra of the reduced Ni4Fe1/(Mg, Al)O_*x*_ show that the average oxidation state of Ni and Fe in the reduced sample was between 0 and +2 (Figure S6). The fitting of the Fourier transforms of the Ni K-edge and Fe K-edge extended X-ray absorption fine structure (EXAFS) spectra of reduced Ni4Fe1/(Mg, Al)O_*x*_ is presented in Table S5 and Figure 5. The Ni edge EXAFS fitting implies a dominant metallic character of Ni with Ni–Ni(Fe) scattering paths with the estimated first shell coordination number of 6.1 ± 1.4. A minor contribution from the Ni–O scattering path was also observed, suggesting the presence of a small fraction of oxidized Ni. Similar findings were also reported for the state of Fe when fitting the Fe K-edge EXAFS data, which demonstrated a dominant contribution from metallic Fe–Fe(Ni) scattering and a minor contribution from Fe–O. A comparison of the fitted values of the Ni–O and Fe–O coordination numbers revealed that Ni had a higher degree of reduction than Fe, which was consistent with the higher oxophilicity of Fe.

The dominant metallic states of Ni and Fe in the reduced catalyst agreed well with the results of TPR and XPS analyses. A visual examination of the Fourier transform of the EXAFS spectra of the reduced Ni4Fe1 sample (Figure S7) indicated that the Fe edge spectrum followed the pattern of the Ni foil (with a fcc crystal structure) rather than the Fe foil (with a bcc structure). This also supports the hypothesis that Fe and Ni formed an alloy with a fcc structure in the reduced Ni4Fe1/(Mg, Al)O_*x*_. The fitted values of the Fe–Fe(Ni) coordination number were slightly higher than those of Ni–Ni(Fe) (Table S5), which may indicate some form of enrichment of Ni on the surface and Fe in the subsurface layers of the alloy NPs.

### Catalytic Activity Evaluation

**Figure 6:**
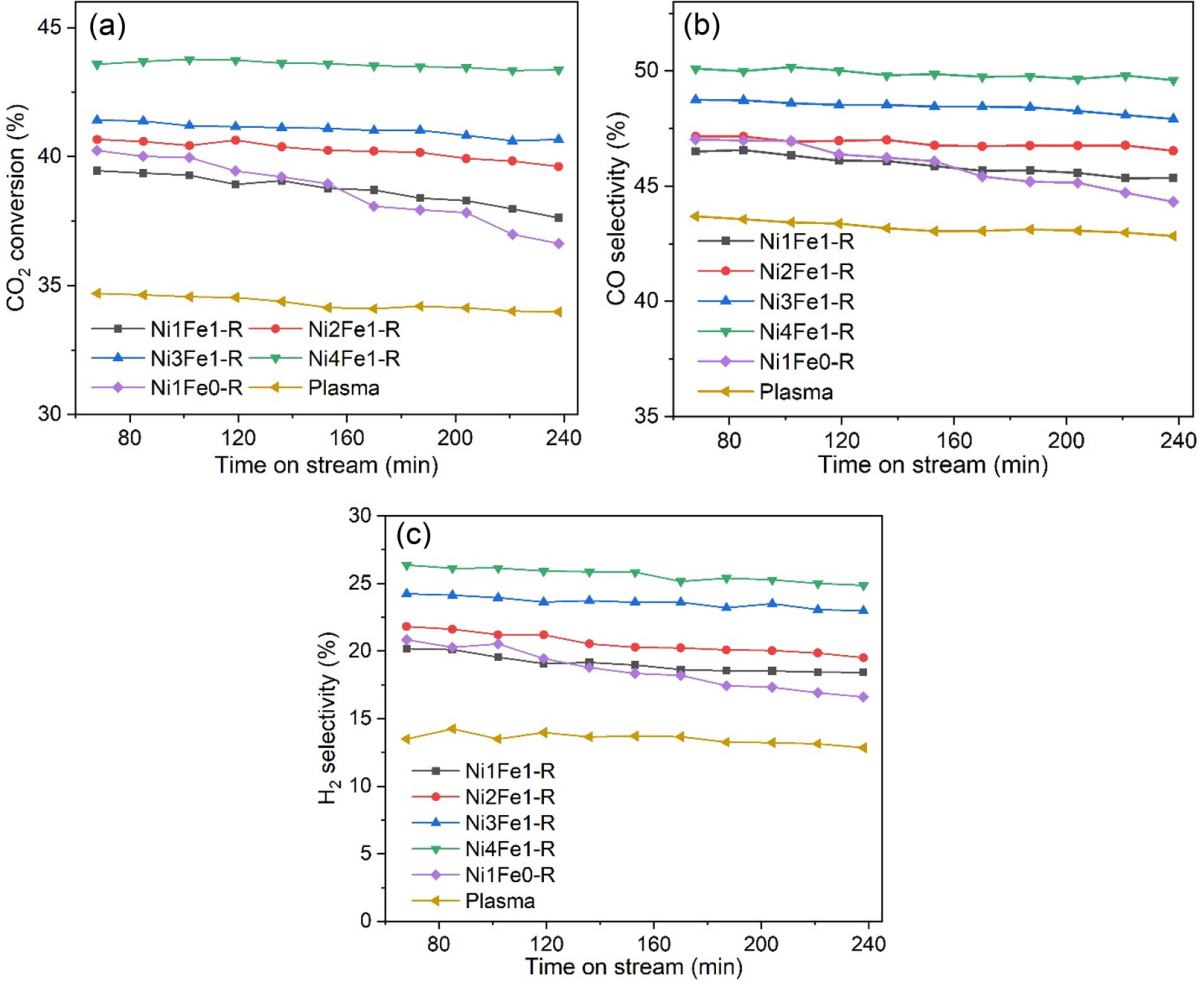
(a) Conversion of CO_2_, selectivity of (b) CO and (c) H_2_ over the Ni–Fe/(Mg, Al)O_*x*_ catalysts with different Ni/Fe ratios during the plasma-catalytic CRT reaction (reaction conditions: 200 mg of catalyst weight diluted with 300 mg of inert SiO_2_, CO_2_/toluene/Ar 7:1:63, and discharge power 36 W).

The low-temperature performance of LDH-derived Ni–Fe/(Mg, Al)O_*x*_ catalysts with various Ni/Fe ratios was tested for CRT in a continuous DBD plasma reactor at 36 W, as shown in Figure 6. Toluene was almost completely converted (>99.6%, as shown in Figure S8), whereas CO_2_ conversions were much lower (34–44%), which was far from the theoretical consumption ratio of 1:1 calculated by eq 3. This phenomenon could be explained by the fact that a plasma system has a distinct reaction mode compared to a thermal system. In a plasma, gas molecules can be activated into vibrationally and electronically excited molecules, atoms, ions, photons, and free radicals by electron impact reactions, allowing the reaction energy barrier to be overcome without significantly increasing the bulk gas temperature.^47^ The formed reactive species will further recombine to form new products via free radical chain reactions.^48^ As a result, toluene with a lower bond dissociation energy (3.7 eV for the C–H bond in methyl, 4.3 eV for the C–H bond in an aromatic ring, 4.4 eV for the C–C bond between the methyl group and the aromatic ring, 5.0–5.3 eV for the C–C bond in an aromatic ring, and 5.5 eV for the C=C bond in aromatic ring) is more easily converted than CO_2_ (5.5 eV) with the aid of plasma discharge. Similar results were reported where CH_4_ with a bond dissociation energy of 4.5 eV generally undergoes higher conversions than CO_2_ during the plasma dry reforming of methane.^49^

The addition of active catalysts in the discharge gap of the DBD plasma significantly enhanced the conversions of CO_2_, together with the selectivity of CO and H_2_, indicating a clear synergistic effect between the DBD plasma and the catalyst. This finding could be attributed to the enhanced heterogeneous surface chemistry induced by plasma-catalyst interactions.^8^ It has also been reported that plasma-induced heating of the catalyst cannot be negligible, especially at a high specific input energy (SIE).^50^ In this case, the plasma-catalysis synergy might be explained by thermal catalysis. However, in our work, the temperature of the catalyst bed during the reforming reaction was around 200 °C at an SIE of 21.6 kJ/L, which is significantly lower than the temperatures (>600 °C) required for the thermal CRT reaction. The less prominent heating effect of plasma during CRT reaction can be explained by the strong endothermic character of the CRT reaction and rapid heat loss by conduction. We tested the thermal-catalytic performance of Ni4Fe1-R at 200 °C, which showed negligible catalytic activity at such a low temperature. Therefore, the thermal effect on the catalyst activity is limited in plasma-catalyzed CRT reaction. Despite the well-known superior activity of Ni atoms for the dissociation of C–C and C–H bonds in toluene, the Ni1Fe0-R catalyst demonstrated poor activity and stability.^51^ In one aspect, the lower surface area and larger Ni particles of Ni1Fe0-R provided fewer active sites for the adsorption and activation of reactants and intermediates. On the other hand, the lower surface area and lesser developed pore structures played a less significant role in enhancing the electric field in plasma (Figure S9), resulting in a weaker plasma-catalysis synergy.

Different from the deactivation mechanism of Ni-based catalysts by coke deposition and Ni sintering during thermal-catalytic reactions, the deactivation of Ni1Fe0-R during the plasma reaction was more likely caused by the phase transformation of metallic Ni induced by plasma discharge, as evidenced by the XRD and FETEM of the spent catalyst in Figures 1 and S10f,g. It has been proven that Ni(111), as the principal exposed facet, was the dominant active site for tar or H_2_ adsorption for subsequent reactions on the catalyst surface, as well as CO_2_ decomposition to adsorbed CO.^51^ However, it is worth noting that partial fcc Ni with a principally exposed facet of Ni(111) (PDF#70-0989) was changed to hexagonal close-packed (hcp) Ni with a principally exposed facet of Ni(011) (PDF#45-1027) after the plasma reaction, which could be responsible for the gradual deactivation of the Ni1Fe0-R catalyst.^52^ The presence of the hcp Ni was further confirmed by the FETEM image of Ni1Fe0-S, which clearly shows the typical lattice fringes of the Ni(011) facet with a spacing of 0.203 nm and the Ni(010) facet with a spacing of 0.230 nm (Figure S10g). In addition, even though the temperature of the bulk gas in plasma remained much lower than that of thermal (catalytic) processes, the agglomeration of Ni particles was observed in Figure S10e. A possible reason could be the presence of local hot spots created by the enhanced electric fields of plasma at the irregular surface of the catalyst or contact points between the catalyst particles.

The integration of Fe by partially replacing Ni atoms contributed to the superior catalytic activity and stability of the NixFe1-R catalysts by forming NiFe alloy NPs. No obvious phase transformation and only minor particle agglomeration could be identified in the XRD patterns and FETEM images of the NixFe1-S catalysts, respectively, (Figures 1c and S10a–d). It is interesting to note from the EDX mapping results that the active Ni and Fe were even more homogeneously dispersed on the support in the Ni4Fe1-S catalyst. A similar result was reported by Jia et al., showing that plasma treatment significantly improved Ni dispersion.^53^

**Figure 7:**
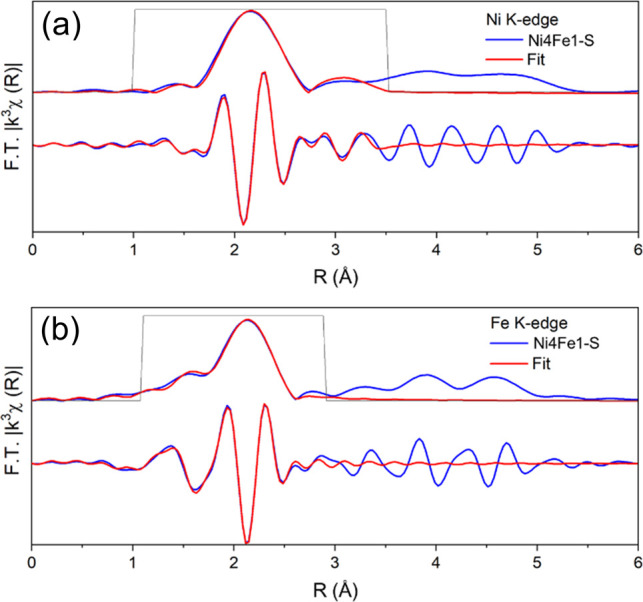
Magnitude and imaginary part of the *k*^3^-weighted Fourier transform of (a) Ni K-edge EXAFS spectrum and fit of the Ni4Fe1-S catalyst (Fourier transform was taken over *k* = 3.3 to 11, and the fitting was done from *R* = 1 to 3.5. The amplitude reduction factor was calculated to be 0.79 from fitting the EXAFS spectrum for the Ni foil) and (b) Fe K-edge EXAFS spectrum and fit of Ni4Fe1-S (Fourier transform was taken over *k* = 3 to 12, and the fitting was done from *R* = 1.1 to 2.9. The amplitude reduction factor was calculated to be 0.7 from fitting the EXAFS spectrum for the Fe foil).

Despite the stability of the NiFe alloy in maintaining the crystalline phase composition and particle size, the partial dealloying of Fe due to its oxidation to FeO_*x*_ during plasma-catalytic CRT reactions can be confirmed by the alloy composition in spent catalysts, as determined by XRD analysis (Table 1). The Ni/Fe ratios in spent catalysts increased from 1.14–3.96 to 1.76–4.13, suggesting the segregation of Fe and enrichment of Ni in NiFe alloys, which is also confirmed by the XPS and EXAFS analyses of spent catalysts. Figure S11 and Table S4 show that the surface Ni^0^ content of spent catalysts (57.3–68.2%) did not differ significantly from that of reduced catalysts (59.6–66.3%). However, the surface Fe^0^ was oxidized to Fe^2+^ with an apparent decrease in Fe^0^ content from 19.1–41.7 to 9.7–29.7%, accompanied by an increase in Fe^2+^ content from 22.8–29.8 to 40.7–65.2%. Among them, Ni4Fe1-S had the highest Fe^0^ content, which correlates with its highest catalytic activity and stability. Similarly, the Fourier transform of the Ni K-edge EXAFS spectrum of the Ni4Fe1-S catalyst after the reaction can be fitted using metallic Ni–Ni(Fe) scattering paths (Figure 7 and Table S6). The data show no evidence of Ni–O interaction, and attempts to fit an additional Ni–O scattering path yielded unrealistic fitting parameters. A comparison of the EXAFS spectra of the reduced and spent samples indicated that Ni was completely reduced during the reaction (no Ni–O scattering path in the Ni4Fe1-S) and that the average particle size of NiFe NPs increased, as indicated by an increase in the Ni–Ni(Fe) coordination number. On the other hand, the fraction of oxidized Fe in Ni4Fe1/(Mg, Al)O_*x*_ increased slightly after the reaction compared to the reduced sample (the fitted coordination number of the Fe–O path increased after the reaction, relative to the change in the metallic Fe–Fe(Ni) path). The Fe edge XANES spectra (Figure S6) also show a slight increase in the oxidation of Fe during the reaction. This can be attributed to partial oxidation of Fe by CO_2_ in the reaction mixture due to the high oxophilicity of Fe, which could potentially play a significant role in coke elimination during tar reforming on the catalyst. Therefore, it is proposed that toluene was activated and decomposed over NiFe particles to generate H_2_ and carbon-bearing intermediates, while the presence of CO_2_ oxidized Fe, leading to the segregation of Fe from the NiFe alloy to form FeO_*x*_. Subsequently, oxygen was transferred from the FeO_*x*_ lattice to a nearby Ni atom, oxidizing the carbon intermediates to CO.^54^

Based on the above analysis, the enhanced performance of NiFe alloy NPs could be explained by the following aspects. The FETEM results in Figures S5a–c and 3d confirmed that NiFe alloy NPs had much smaller particle sizes with higher dispersion, providing more efficient and stable active sites to overcome the catalytic energy barriers of CRT in plasma-involved interfacial catalysis.^55^ At the same time, the enrichment of Fe species on the surface of the NiFe alloy accelerated the adsorption of CO_2_ and O-related intermediates (e.g., O and OH) by providing more oxygen vacancies (as demonstrated by the CO_2_-TPD result in Figure 2) and thus the oxidation of carbon intermediates to CO.^9,56^ Furthermore, the increased surface area and more uniform morphology caused by Fe doping promoted the adsorption and transfer of reactants and intermediates, as well as strengthening the microdischarge of plasma inside the developed channels and pores of Ni–Fe/(Mg, Al)O_*x*_ catalysts. Among the various catalysts, Ni4Fe1-R showed the highest catalytic activity and stability for plasma-enabled CRT, which should be attributed to its highest surface area and strongest lattice distortion. Excess Fe addition decreased the catalytic activity since Fe was less reactive than Ni.^57^ In addition, the catalysts with a higher Fe loading showed weaker Ni–Fe interactions, where Fe was easier to segregate from the alloy to form FeO_*x*_, resulting in catalyst deactivation.

To better understand the advantages of the plasma-catalytic system, the performance of the catalysts was also tested in a conventional thermal system and compared to that of the plasma-catalytic system, as illustrated in Figure S12. The catalytic performance in the thermal CRT reaction followed a trend similar to that in the plasma-catalytic reaction, that is, Ni4Fe1-R > Ni3Fe1-R > Ni2Fe1-R > Ni1Fe1-R > Ni1Fe0-R. Ni4Fe1-R also achieved the highest activity and stability in thermal-catalytic reactions, which corresponded to its physiochemical properties. However, it is noteworthy that the difference in catalytic performance between various catalysts, especially between NiFe alloy catalysts and Ni-based catalysts, was more pronounced in the thermal process than in the plasma process. This phenomenon can be explained by the distinct mechanisms in thermal catalysis and plasma catalysis. In thermal-catalytic processes, the reaction performance is strongly dependent on the activation ability of the catalyst, which plays a crucial role in lowering the activation barrier of the reaction. However, in a plasma-catalytic system, the plasma has inherent activity in igniting and accelerating the reaction. In comparison, the role of the catalyst could be less significant. When comparing the CRT performance in plasma-catalytic and thermal-catalytic systems, it was found that even at a high temperature of 600 °C, the conversions of toluene and CO_2_ in the thermal-catalytic system were much lower than those in the plasma-catalytic system due to the thermodynamic barrier. Furthermore, a more severe coke deposition can be observed on spent catalysts in the thermal process. As shown in Figure S13a, the mass loss of spent catalysts for the plasma-catalyzed CRT reaction ranged from 30.8 to 36.9%. The mass loss was caused primarily by the oxidation removal of carbon-containing intermediates deposited on the catalysts that could not be vaporized at low temperatures, as indicated by a broad exothermic peak between 150 and 550 °C in differential scanning calorimetry (DSC) curves. The small shoulder peak at around 600 °C could be ascribed to coke formation, which was much less pronounced than the thermal process, as shown in Figure S13b. The superior coke resistance ability of the plasma-catalytic process was further confirmed by the FETEM results depicted in Figure S10, where no obvious coke was detected on spent catalysts. However, the selective formation of target products was promoted by the increased selectivity of H_2_ (>60%) and CO (>65%) in the thermal process. The relatively lower selectivity of H_2_ and CO in the plasma-catalytic system, as the most dominant limitation, might be attributed to the limited plasma-catalysis synergy since the catalyst was only packed in a small region of the discharge zone, leaving a large portion of the plasma discharge volume without any catalyst. As a result, undesirable byproducts such as CH_4_ (<2%) and light hydrocarbons (<1%) were produced in the plasma zone without catalyst packing. A detailed element balance is provided in Figure S14 and Table S7 in the Supporting Information. Further efforts should be made to maximize the plasma-catalysis synergy, either by optimizing the plasma catalysis configurations and discharge parameters or by developing functionalized catalysts with high activity and selectivity. In addition, the effect of CO_2_ concentration on the performance of the plasma-catalyzed CRT reaction was also investigated in this study, with relevant results presented in Supporting Information (Figures S15 and S16).

### Mechanism of Plasma-Catalytic CRT Reaction

Our results show that the presence of LDH-derived Ni–Fe/(Mg, Al)O_*x*_ catalysts, particularly Ni4Fe1-R catalyst, significantly promoted the plasma-catalyzed CRT reaction. To gain more insights into the plasma-enhanced surface reactions, in situ FTIR was performed over the Ni4Fe1-R and Ni1Fe0-R catalysts, as shown in Figures S17, S18, and 8. Table S8 summarizes the detailed information about the IR bands of surface-adsorbed intermediates. In Figure S17, the characteristic IR bands at 3075 and 3037 cm^–1^ are assigned to the υ(C–H) stretching vibration of the aromatic ring, while the bands at 1075 and 1030 cm^–1^ belong to the in-plane υ(C–H) bending of the aromatic ring. Moreover, the typical skeleton υ(C=C) vibrations of the toluene aromatic ring can be observed at 1612, 1499, and 1459 cm^–1^, suggesting that toluene can be efficiently adsorbed on the surfaces of both Ni4Fe1-R and Ni1Fe0-R.^58^ However, it is interesting to find that the characteristic bands at 2937 and 2881 cm^–1^ may be assigned to the symmetric or asymmetric υ(C–H) stretching of methylene (−CH_2_) instead of methyl (−CH_3_) with characteristic bands in the range 2970–2950 cm^–1^, indicating the absence of −CH_3_ groups in the intermediate species adsorbed on the catalyst surface. The bands at 1420 cm^–1^ corresponding to the bending vibration of −CH_2_ further confirm the formation of methylene.^59^ Meanwhile, a typical υ(C–O) vibration band can be found at 1041 cm^–1^, which might be attributed to the reaction of toluene with the lattice oxygen of the catalyst (*O) to form benzoyl oxide species (C_6_H_5_–CH_2_–O).^60^ Furthermore, weak bands associated with asymmetric (1540 cm^–1^) and symmetric (1390 cm^–1^) υ(C=O) stretching vibrations also appear, indicating the formation of a small amount of benzoate species (C_6_H_5_–COO).^58^ Therefore, it can be deduced that the first step of toluene adsorption is the cleavage of the C–H bond in the −CH_3_ group of toluene. The abstraction of a hydrogen atom from toluene results in the formation of benzyl species, which are then attached to the catalyst surface by reacting with the *O to form benzyl alcoholate species.^61^ At low temperatures, partial benzoyl oxide species can further interact with active oxygen species to produce benzoate species.^58^

**Figure 8:**
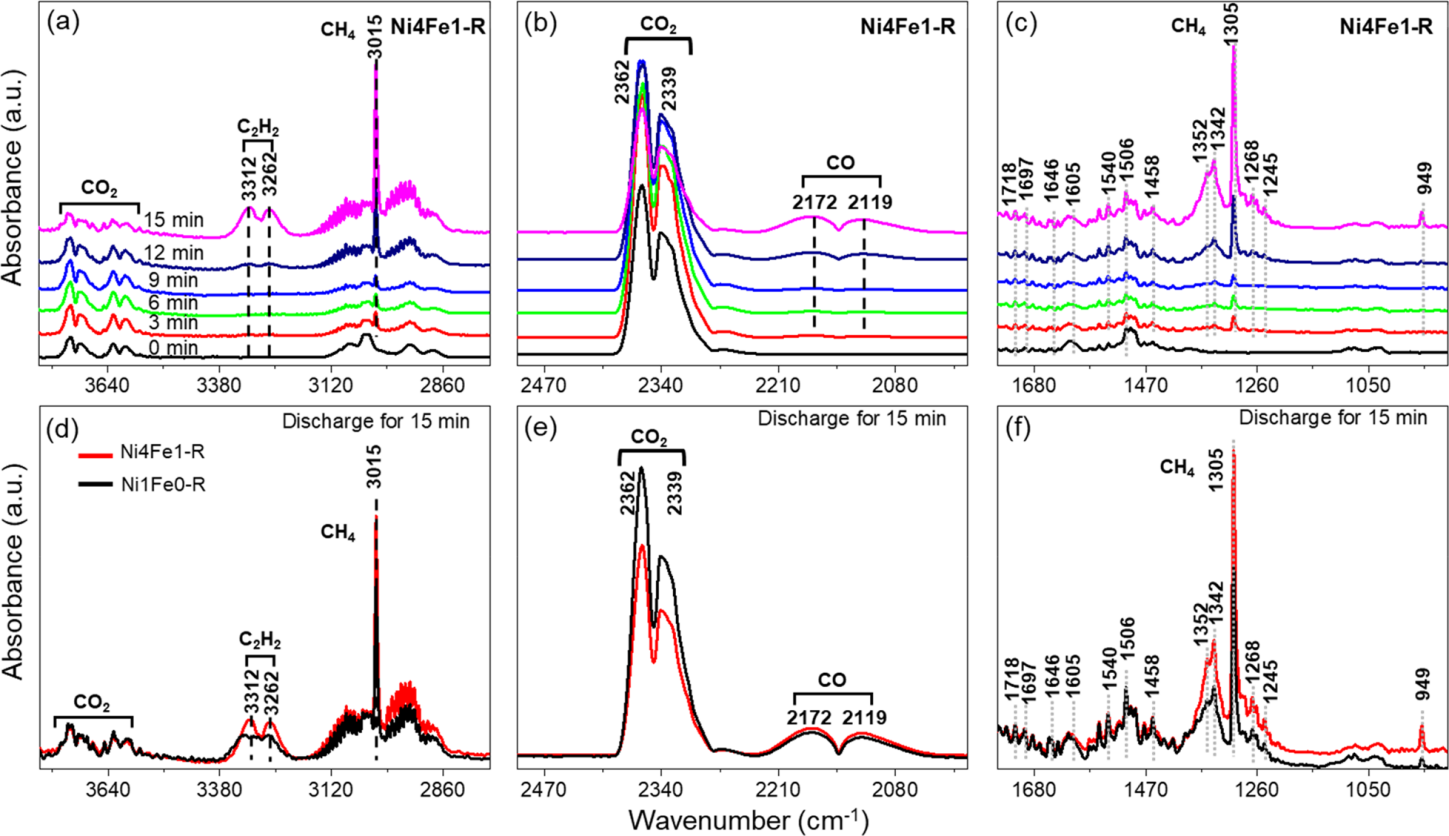
(a–c) In situ FTIR spectra of plasma-catalytic CRT over the Ni4Fe1-R catalyst at 25 °C (discharge time 15 min, feed gas 10 vol % CO_2_/1.5 vol % C_7_H_8_ diluted in argon, total flow rate 20 mL/min) and (d–f) comparison of in situ FTIR spectra over the Ni4Fe1-R and Ni1Fe0-R catalysts at a discharge time of 15 min.

After purging the cell with a mixture of CO_2_ and Ar for 5 min, the characteristic bands of gaseous CO_2_ molecules at 2362 and 2339 cm^–1^ emerge, as well as the CO_2_ overtone bands at 3700 and 3600 cm^–1^.^62,63^ Additionally, the appearance of IR bands of OH vibration at 3730 and 3630 cm^–1^ (Figure 8a) confirms the adsorption of CO_2_ as a bicarbonate species (*HCO_3_) over the hydroxyl groups of the (Mg, Al)O_*x*_ support.^62^ Nevertheless, the characteristic IR bands of monodentate carbonate *CO_3_ are absent, possibly due to a lack of available *O sites that have been occupied by toluene. It was reported that for Ni–Fe/(Mg,Al)O_*x*_ catalysts, surface *OH species with weak basic sites would facilitate the formation of *HCO_3_, whereas *O species would provide strong basic sites to produce monodentate *CO_3_. However, *HCO_3_ played a more significant role in CO_2_ decomposition compared to *CO_3_, which was more stable and difficult to remove even at high temperatures.^64^

**Figure 9:**
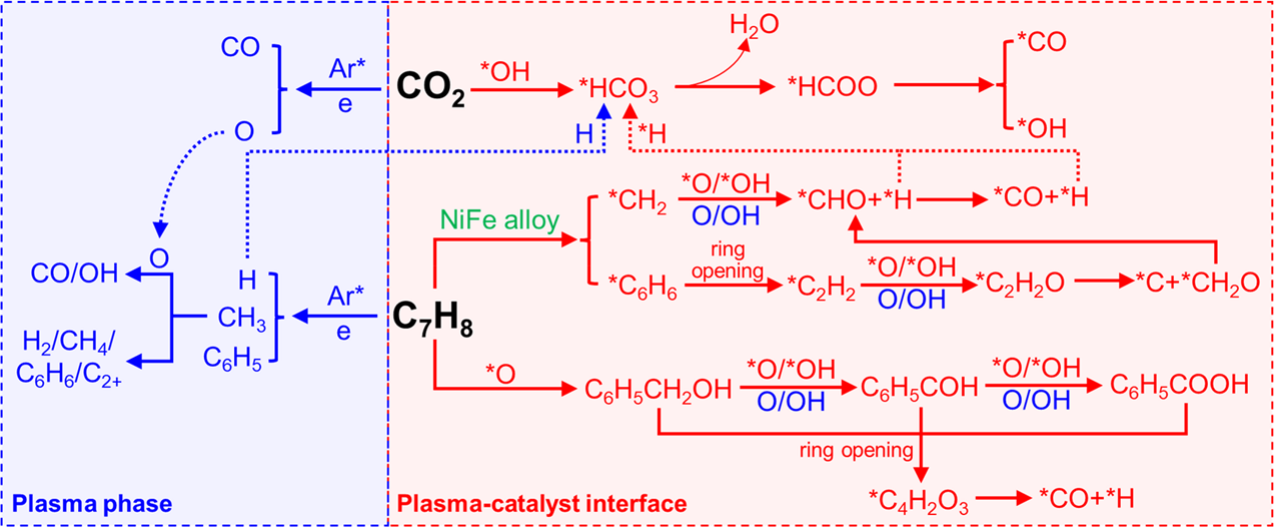
Proposed reaction pathway of plasma-catalytic CRT reaction over LDH-derived Ni–Fe/(Mg, Al)O_*x*_.

When switching on the plasma, the intensities of the CO_2_ and toluene peaks gradually decrease and even disappear, while some new peaks appear, implying that CO_2_ and toluene underwent a sufficient reaction on the plasma–catalyst interface (Figure 8). The characteristic absorption bands of gaseous CH_4_ (3015 and 1305 cm^–1^) and CO (2172 and 2119 cm^–1^) confirm the formation of CH_4_ and CO as major products of CRT, respectively.^63,65^ A small number of short-chain olefins were also generated with characteristic absorption bands at 949 cm^–1^. Moreover, abundant IR bands related to the reaction intermediates can be observed, including benzyl alcoholate (1458, 1352, and 1245 cm^–1^),^66,67^ benzaldehyde (1697 and 1646 cm^–1^),^68^ and benzoate species (1540, 1342, and 1268 cm^–1^),^67^ which were generated from the oxidation of toluene by reactive oxygen species (Figure 8c). Meanwhile, some important ring-opening intermediates could also be detected, such as C_2_H_2_ at 3262 or 3267 cm^–1^^69^ and surface maleate species at 1718 and 1506 cm^–1^.^68,70^ Furthermore, the formation of these crucial intermediates, such as benzene, maleic anhydride, benzaldehyde, and benzyl alcohol, can also be evidenced by the gas chromatography–mass spectrometry (GC–MS) analysis in Figure S19a. Accordingly, the reaction pathway in the plasma-catalytic CRT reaction is proposed in Figure 9. In the plasma phase without a catalyst, CO_2_ is activated by excited Ar and electrons by transferring their energy in collisions and decomposing to CO and O*, whereas toluene is converted into a variety of active species including H, CH_3_, and C_6_H_5_. The generated active species will then recombine to form H_2_, CH_4_, light hydrocarbons (C_2_–C_4_), C_6_H_6_, and even higher hydrocarbons. However, the mechanism of CRT will be significantly altered at the plasma–catalyst interface. Firstly, CO_2_ is adsorbed by the hydroxyl groups (*OH) on the (Mg, Al)O_*x*_ support to form *HCO_3_, which then reacts with gaseous H radicals generated by the plasma and adsorbed *H on the catalyst to generate the formate species (*HCOO, 1605 cm^–1^). The formate species can be directly decomposed into CO and *OH. Meanwhile, toluene could be adsorbed and cracked to *C_6_H_6_ and *CH_2_ on the surface of the Ni–Fe alloy, and then *C_6_H_6_ is further decomposed to *C_2_H_2_ species via a ring-opening reaction, according to the in situ FTIR results. Following that, the adsorbed *CH_2_ and *C_2_H_2_ species will react with highly reactive oxygen species (O and OH radicals generated by plasma, and adsorbed *O and *OH species) to form *CHO, which will then be decomposed into CO and adsorbed *H species. In addition, another route of toluene decomposition may exist based on the in situ FTIR results. Specifically, toluene is adsorbed over the lattice oxygen or adsorbed oxygen (*O) to form benzyl alcoholate species, which are then oxidized to benzaldehyde and benzoate species. These aromatic intermediates will go through a ring-opening process, generating maleate species before being decomposed to CO and adsorbed *H. The adsorbed *H can be terminated by producing H_2_ gas.

The evolution of typical surface intermediates over the Ni4Fe1-R and Ni1Fe0-R catalysts shows no obvious difference when comparing the in situ FTIR results (Figures 8 and S18). However, it should be noted that the intensity of toluene adsorption over the Ni4Fe1-R catalyst is higher than that over the Ni1Fe0-R catalyst, which should be attributed to the higher lattice distortion and greater oxygen vacancies of Ni4Fe1-R, as confirmed by the XPS result (Figure 4c). Furthermore, the lower intensity of CO_2_ and higher intensity of CO over Ni4Fe1-R after switching on the plasma for 15 min suggest its superior catalytic performance in the plasma-catalyzed CRT reaction, which is supported by the higher amount of reaction intermediates on its surface. It is due to the higher reactivity of the NiFe alloy in activating the reactants, as well as the more abundant active oxygen species in the Ni4Fe1-R catalyst, which facilitates the adsorption and oxidation of intermediates. This hypothesis could be evidenced by the formation of more oxygen-containing intermediates over Ni4Fe1-R than over Ni1Fe0-R (Figure S19).

## Conclusions

In summary, we investigated the plasma-catalytic CO_2_ reforming of toluene over LDH-derived catalysts in a DBD reactor at a low temperature. The ultrathin LDH-derived NiFe alloy catalysts demonstrated promising performance in this system, with a clear synergistic effect between the plasma and the catalyst. Among the catalysts, the Ni1Fe0-R catalyst exhibited the poorest catalytic performance due to its low specific surface area and large particle size. In addition, both the phase transformation of Ni from a fcc Ni to a hcp Ni and the agglomeration of Ni particles caused by hot spots induced by plasma contributed to the deactivation of Ni1Fe0-R. The incorporation of Fe favored the enhancement of catalyst performance by generating NiFe alloys with a smaller particle size and higher dispersion, which provided abundant and stable active sites for toluene cracking to H_2_ and carbon intermediates. In addition, the enrichment of Fe on the surface of the NiFe alloy accelerated the adsorption of CO_2_ by providing more oxygen vacancies. The Ni4Fe1-R catalyst achieved the most superior performance due to its large surface area and strong lattice distortion. The in situ FTIR characterization of the plasma-catalytic CRT reaction reveals that the Ni4Fe1-R catalyst promoted both toluene adsorption and oxidation when compared to the Ni1Fe0-R catalyst, with more oxygen-containing intermediates produced. This work provides new insights into the mechanism of the plasma-catalytic CO_2_ reforming of toluene, which is critical for the development of more efficient plasma-catalytic systems for the synthesis of fuels and chemicals.

## Experimental Section

### Synthesis of LDH-Derived Catalysts

The Ni–Fe–Mg–Al LDH precursors with various Ni/Fe ratios (1:1, 2:1, 3:1, 4:1, and 1:0) were synthesized using a pH-controlled coprecipitation method. Specifically, hydrated metal nitride salts, including Ni(NO_3_)_2_·6H_2_O, Fe(NO_3_)_3_·9H_2_O, Mg(NO_3_)_2_·6H_2_O, and Al(NO_3_)_3_·9H_2_O (Macklin), were dissolved completely in 200 mL of deionized water. The metal nitride solution and Na_2_CO_3_ aqueous solution (1 M) were added dropwise into a beaker containing 15 mL of preheated deionized water simultaneously. In addition, NaOH aqueous solution (2 M) was also added into the mixture at the same time to maintain the pH of the solution at 10. The mixture was stirred vigorously to ensure sufficient mixing and precipitation, and then the resulting suspension was aged at 60 °C for 18 h. The precipitate was collected by centrifugation at 8000 rpm and then washed several times with deionized water and dried at 105 °C overnight. The obtained LDH precursors with various Ni/Fe ratios were denoted as NixFey-HT. The molar and mass amounts of starting materials for preparing the precursors are listed in Table S1. To ensure a constant content of active metals (Ni and Fe) for different catalysts, the molar ratio of (Ni^2+^ + Fe^3+^) to (Mg^2+^ + Al^3+^) was kept at 3:14. Moreover, the ratios of (Fe^3+^ + Al^3+^)/(Fe^3+^ + Al^3+^ + Ni^2+^ + Mg^2+^) were maintained at 0.24–0.32 since the M^3+^/(M^3+^ + M^2+^) ratio of 0.17–0.33 is more favorable for forming a complete LDH structure. To obtain the LDH-derived metal oxides, all LDH precursors were calcined at 600 °C for 6 h in stacked air at a ramping rate of 10 °C/min, which was denoted as NixFey-F.

### Performance Test of the Plasma-Catalytic CRT Reaction

CRT was performed in a hybrid DBD plasma-catalytic reactor at atmospheric pressure and low temperature, as illustrated in Figure S1. The DBD plasma reactor consists of a threaded stainless high-voltage electrode (O.D. = 16 mm), a quartz tubular dielectric (I.D. = 20 mm, thickness: 2.5 mm, and length: 360 mm), and a wire-mesh ground electrode (length: 150 mm) that was wrapped over the quartz tube. As a result, the discharge length, gap, and volume were 150 mm, 2 mm, and 16.95 mm^3^, respectively. A variable and high-frequency plasma generator (CTP-2000K, Coronalab, Nanjing, China) with a maximum peak-to-peak voltage of 30 kV and a center frequency of 10 kHz served as the AC power supply of the DBD plasma. The electrical signals were monitored by a digital oscilloscope (DS2102A, Rigol Technologies, China).

Prior to the activity test, the catalysts were prereduced at 800 °C for 1 h in H_2_ (20 mL/min), and the samples were labeled as NixFey-R. Each catalyst (200 mg diluted with 300 mg of inert SiO_2_) was sandwiched between quartz wool and packed in the center of the discharge region. The average discharge power of the DBD reactor was 36 W, which was calculated using the Lissajous method. The temperature of the plasma-catalyst zone was ∼200 °C at 36 W, measured using an optical fiber thermometer (Omega, FOB102). C_7_H_8_, a model tar compound, was sampled with a syringe pump (Longer LSP01-1A, China) at a speed of 6.78 μL/min. A mixture of toluene (∼1.4 mL/min as a gas component), CO_2_ (10 mL), and Ar (88.6 mL/min) with a total flow rate of 100 mL/min was preheated and fed into the DBD reactor. The C_7_H_8_/CO_2_ molar ratio was 1:7, which was the stoichiometric ratio of the CRT reaction in eq 3. The temperatures of the preheater, plasma-catalytic reactor, and postheater were maintained at 200 °C in order to avoid the condensation of gaseous toluene and products. In addition, the performance of the CRT reaction using various CO_2_ concentrations (0, 5, 10, 15, 20, 25, and 30%) was also investigated at a constant total flow rate of 100 mL/min. The detailed analysis procedures for gaseous and liquid products are provided in the Supporting Information.
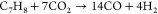
3

The conversion of toluene and CO_2_ and the selectivity of CO, H_2_, and light hydrocarbons LHCs (typically CH_4_ and C_2_–C_4_) were calculated as follows.
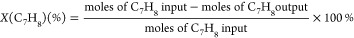
4
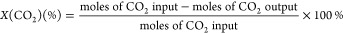
5
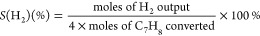
6
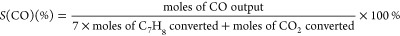
7
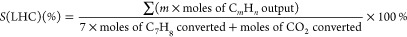
8

### Catalyst Characterization

ICP-OES (Thermo Fisher iCAP PRO, USA) was employed to determine the loadings of Ni, Fe, Mg, and Al in the fresh calcined NixFey-F catalysts. Prior to the analysis, 10 mg of each sample was digested in *aqua regia* (10 mL) at 120 °C for 4 h.

N_2_ physical adsorption–desorption experiments were conducted on a Micromeritics ASAP 2460 adsorption apparatus at −196 °C to determine the specific surface area, cumulative pore volume, and pore diameter distribution of freshly calcined and reduced catalysts. A certain amount of the catalyst was heated at 200 °C for 6 h in vacuum to remove the volatile species adsorbed on the catalyst surface. The specific surface area was calculated from the isotherm by the Brunauer–Emmett–Teller model, while the pore parameters were obtained using the Barrett–Joyner–Halenda model.

The XRD patterns of LDH precursors and fresh calcined, reduced, and spent catalysts were collected by a Rigku Ulitma IV diffractometer (Japan) equipped with Cu Kα radiation at 40 kV and 40 mA. The samples were scanned in the 2θ range of 10–80° at a scanning rate of 10°/min.

H_2_-TPR analysis of fresh calcined catalysts was conducted to characterize the reducibility of catalysts on a Micromeritics ChemiSorb 2720 system equipped with a thermal conductivity detector. In brief, 80 mg of catalyst was pretreated at 200 °C for 30 min in He flow (30 mL/min) to remove the physically absorbed ambient gas molecules before cooling to 50 °C in He flow. Finally, a mixture of H_2_ and N_2_ (5 vol % H_2_, total flow rate 30 mL/min) was passed through the catalyst bed, and the temperature was raised from 50 to 950 °C at a heating rate of 10 °C/min.

CO_2_-TPD was also performed on a Micromeritics ChemiSorb 2720 system to evaluate the basicity of catalysts. Specifically, 100 mg of the catalyst was initially in situ reduced at 800 °C for 1 h in 5 vol % H_2_ diluted by N_2_ (total flow rate 30 mL/min). After cooling to 50 °C in He flow, the reduced catalyst was exposed to the CO_2_ stream (30 mL/min) for 30 min and flushed with He for 30 min to remove the weakly adsorbed CO_2_. The desorption process was measured from 50 to 950 °C at a heating rate of 10 °C/min in He flow (30 mL/min).

The surface morphology and microstructure of LDH precursors and fresh calcined and reduced catalysts were recorded using FESEM (JEOL JSM-7800F, Japan). In addition, TEM images and EDX element maps of reduced and spent catalysts were taken using FETEM (FEI Talos F200X G2, USA). Prior to FETEM analysis, the catalyst was ultrasonically dispersed in ethanol and dropped on a super-thin carbon film. The particle size distribution of active metals was analyzed by ImageJ software, as well as the lattice spacing.

AFM (Dimension Icon, Bruker, USA) was used to determine the topographical information of the as-synthesized LDH precursor. Specifically, the sample was prepared by depositing the ultrasonic solution of LDH precursor onto a mica wafer, and subsequently, the AFM images were recorded in the noncontact mode using Si tips.

XPS analysis of reduced and spent catalysts was performed using a Thermo Scientific K-Alpha+ instrument equipped with an Al Kα X-ray radiation source (*h*υ = 1486.6 eV) at 225 W (15 kV, 15 mA). The raw data were calibrated by using the C 1s lines of adventitious carbon with a binding energy of 284.6 eV.

Ni K-edge and Fe K-edge XANES and EXAFS data of reduced and spent Ni4Fe1/(Mg, Al)O_*x*_ catalysts were acquired on the XAFCA beamline of the Singapore Synchrotron Light Source using transmission mode. At the same time, Ni foil, NiO, Fe foil, FeO, and Fe_2_O_3_ were employed as standard references for energy calibration.

Thermogravimetric (TG) analysis was carried out to evaluate the coke deposition on the surface of spent catalysts using a Mettler Toledo TGA/DSC 1/1100 thermal analyzer. A certain amount of spent catalyst was loaded onto a corundum crucible and heated in air from 50 to 950 °C at a heating rate of 10 °C.

The in situ FTIR of plasma-catalytic CRT over the Ni4Fe1-R and Ni1Fe0-R catalysts was performed using a JASCO FT/IR-4600 spectrometer (transmission mode, resolution of 0.7 cm^–1^) equipped with a Peltier stabilized DLaTGS detector and a self-designed in situ DBD/gas cell where plasma was generated in the catalyst bed. The detailed configuration of the cell can be found in our previous publication.^71^ The catalyst was initially reduced in a H_2_ flow (20 mL/min) at 800 °C for 1 h before being pressed into a thin flake (diameter: 10 mm and thickness: ∼0.3 mm) and loaded into the FTIR cell. The catalyst was heated at 98 °C for 1 h in an Ar flow (99.999%, 100 mL/min) to remove the adsorbed water and gas impurities. Subsequently, the temperature of the catalyst and the flow rate of Ar were reduced to 35 °C and 20 mL/min, respectively, and remained for 20 min to collect the background IR spectra. The saturated toluene vapor (99.9%) was continuously introduced into the cell using a bubbling method at room temperature (30 min) with Ar as the carrier gas (20 mL/min) to achieve an adsorption equilibrium of toluene on the catalyst. The residual toluene in the cell was purged with a mixture of CO_2_ (2 mL/min) and Ar (17.8 mL/min) for 5 min. The plasma was then ignited in a continuous flow of CO_2_ and Ar at a total flow rate of 19.8 mL/min, with spectra collected every 3 min for 9 min. Finally, the gas cell was sealed, and the IR spectra were monitored under plasma discharge for another 6 min. The discharge parameters were identical to those used in the plasma-catalytic activity tests.

## Supporting Information Available

Experimental setup of plasma-catalytic CRT reaction, crystalline phase compositions, chemical compositions, texture properties, morphologies, surface chemical compositions, XAS characterizations, TG-DSC curves and in situ FTIR results of the catalysts, reaction performance, elemental balance, electrical diagnostics, and liquid intermediates of plasma-catalytic CRT reaction (PDF)

## Notes

The data supporting the findings of this study are available from the corresponding authors upon reasonable request.
