# Supplementary material for: Plasma-Catalytic CO2 Reforming of Toluene over Hydrotalcite-Derived NiFe/(Mg, Al)Ox Catalysts
Source: JACS Au. 2023 Feb 17;3(3):785–800. doi: 10.1021/jacsau.2c00603 (PMC10052232; doi:10.1021/jacsau.2c00603)
Supplement: Supplementary file 1 — au2c00603_si_001.pdf [file au2c00603_si_001.pdf]

Plasma-catalytic CO_2 reforming of toluene over hydrotalcite-derived NiFe/(Mg, Al)O_x catalysts

Lina Liu [a], Jing Dai [a], Sonali Das [b,1], Yaolin Wang [c], Han Yu [a,*], Shibo Xi [d], Zhikun Zhang [e,*], Xin Tu [c,*]

[a] College of Environmental Science and Engineering, Ministry of Education Key Laboratory of Pollution Processes and Environmental Criteria, Nankai University, Tianjin 300350, China

[b] Department of Chemical and Biomolecular Engineering, National University of Singapore, 117585, Singapore

[c] Department of Electrical Engineering and Electronics, University of Liverpool, Liverpool, L69 3GJ, UK

[d] Institute of Chemical and Engineering Sciences, A* STAR, 1 Pesek Road, Jurong Island, 627833, Singapore

[e] School of Energy and Environmental Engineering, Tianjin Key Laboratory of Clean Energy and Pollution Control, Hebei University of Technology, Tianjin 300401, China

[1] Present address: Department of Chemical Engineering, Indian Institute of Technology Bombay, Mumbai, 400076, India

[*]Corresponding authors

Detailed procedures for gaseous and liquid product analysis

The gaseous products and toluene were determined by an on-line gas chromatograph (GC, 7890A, Agilent Technologies, USA). Specifically, the permanent gaseous products, including H_2, CO and CO_2 were analyzed using a Carboxen 1000 packed column and a TCD, while light hydrocarbons (LHCs) and toluene were measured using a DB-5 capillary column and a flame ionization detector (FID). The injection valves, ovens, and detectors were maintained at 150 ºC, 80 ºC and 250 ºC, respectively.

The complex liquid byproducts of the plasma CRT reaction with and without the Ni4Fe1-R catalyst were collected with methanol and analyzed using gas chromatography-mass spectrometry (GC-MS, Trace1300-ISQ7000, Thermo Fisher Scientific, USA) equipped with an HP-5MS capillary column (30 m × 0.250 mm, 0.25 μm; Agilent Technologies, USA). 1 μL of sample in methanol was injected using the split sampling mode with a split ratio of 10:1. Helium was used as the carrier gas at a flow rate of 1 mL/min. The temperature of both the ion source and the transmission line remained at 280 ºC. The GC oven was initially set to 50 ºC for 5 min, then programmed to 200 ºC at a rate of 25 ºC/min and held for 5 min, and finally increased to 280 ºC at a rate of 5 ºC/min and held for 5 min. Chromatograms were recorded in full scan mode with a mass range between 35 and 500 amu. Finally, the peaks were identified by comparing their mass spectra to those in the NIST 11 mass spectra library.

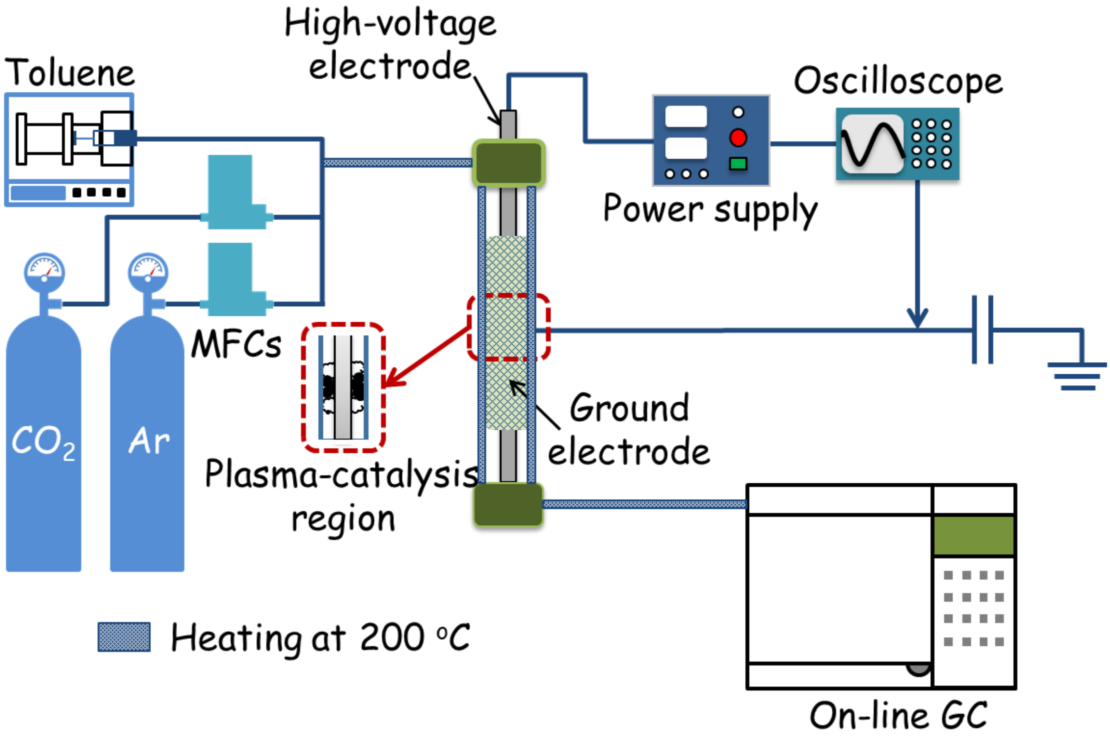

Fig. S1. Schematic diagram of the DBD plasma-catalytic system for CO_2 reforming of toluene

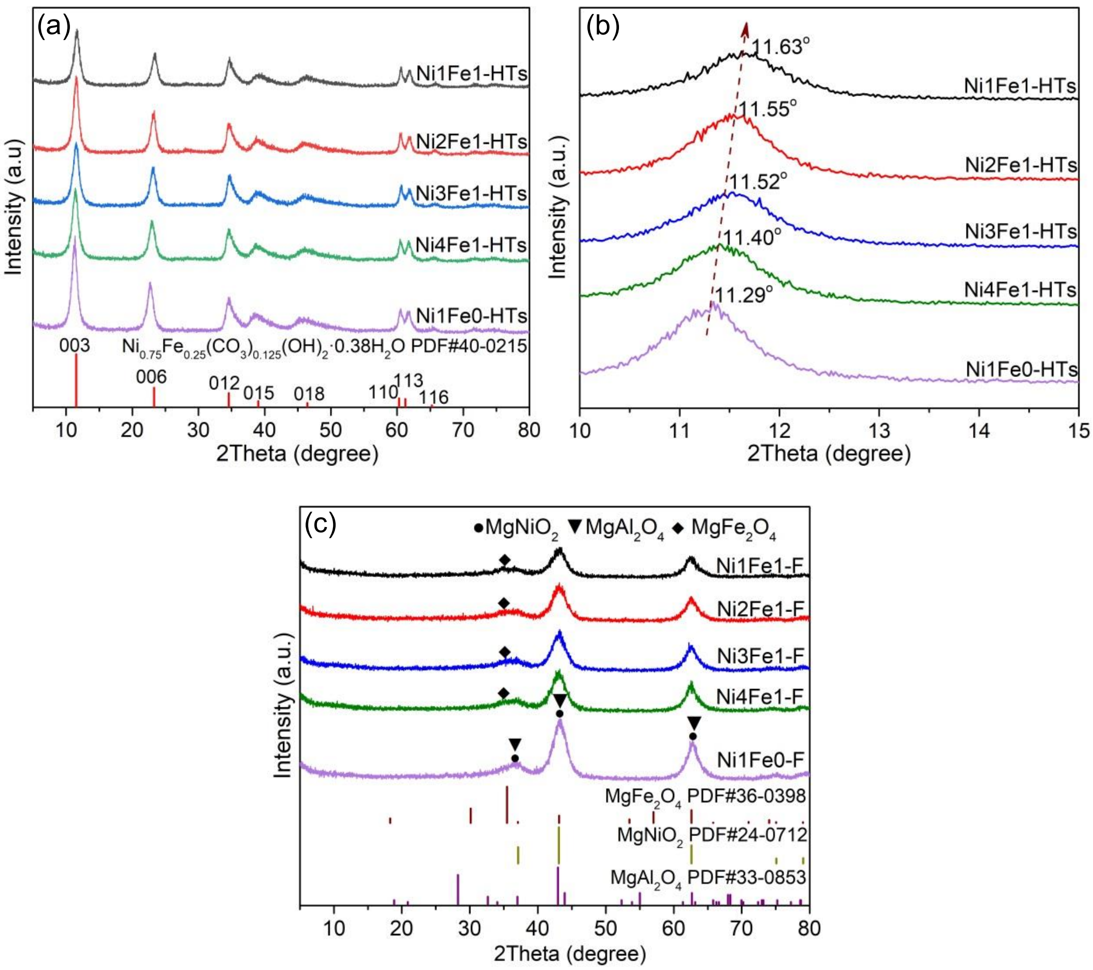

Fig. S2. XRD patterns of (a, b) as-synthesized Ni-Fe-Mg-Al LDH precursor, and (c) fresh calcined LDH-derived catalysts.

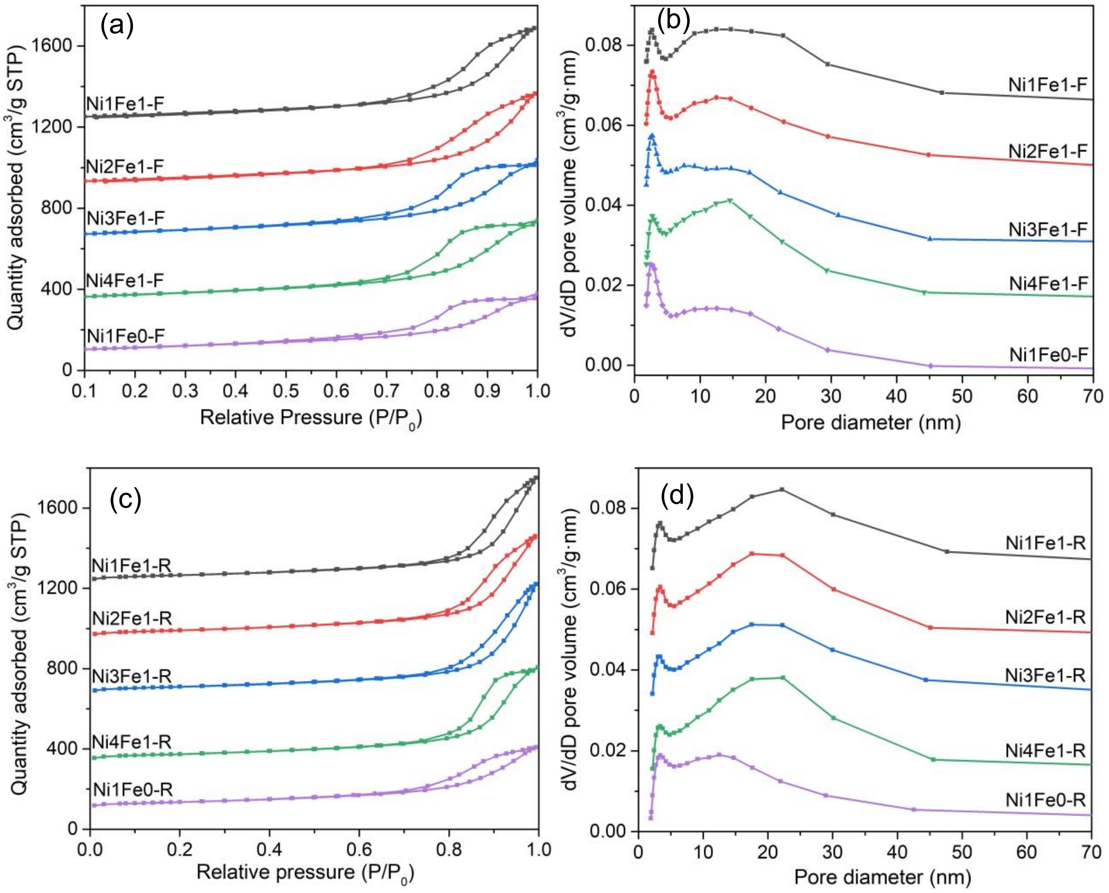

Fig. S3. N_2 adsorption isotherm and pore diameter distribution of (a, b) calcined and (c, d) reduced Ni-Fe/(Mg, Al)O_x catalysts.

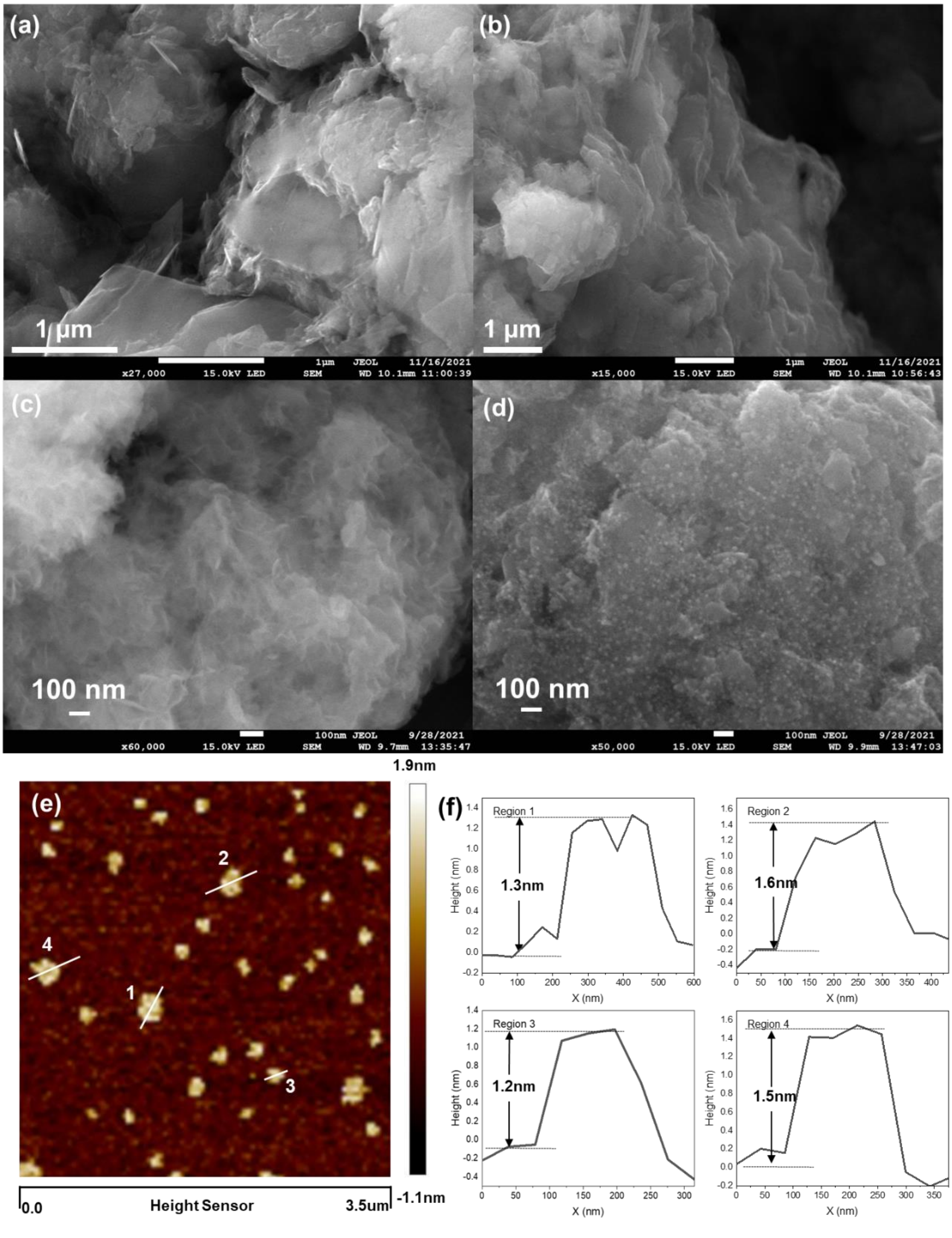

Fig. S4. FESEM images of as-synthesized (a) Ni4Fe1-Mg-Al and (b) Ni-Mg-Al hydrotalcite precursors, (c) calcined and (d) reduced Ni/(Mg, Al)O_x catalysts, (e) AFM image and (f) the height profile of as-synthesized Ni4Fe1-Mg-Al hydrotalcite precursor.

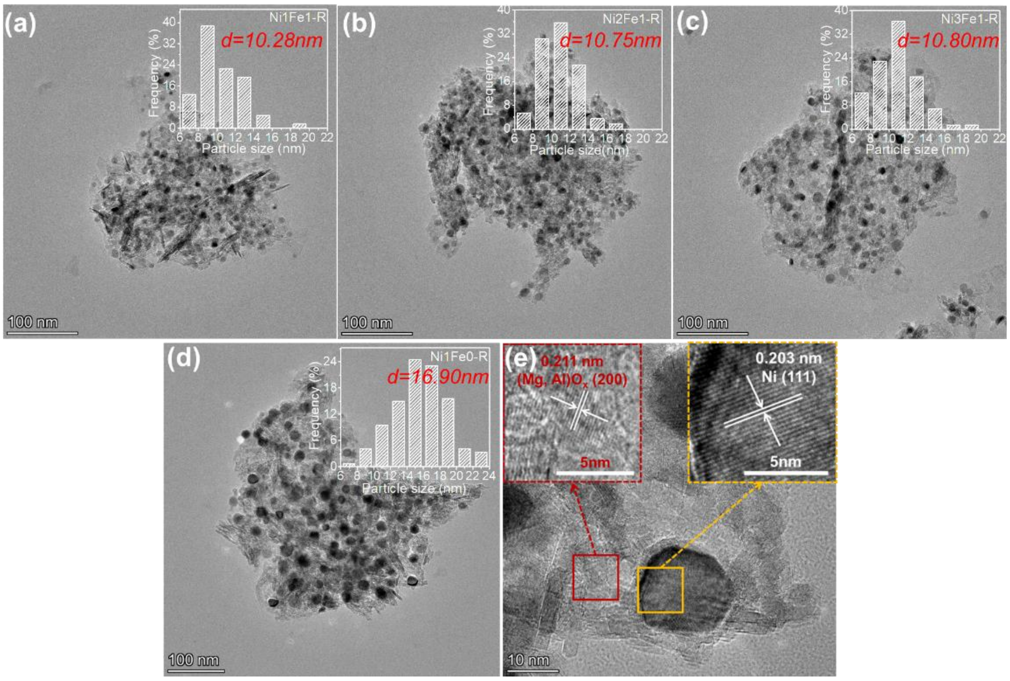

Fig. S5. (a-d) FETEM images and nanoparticle size distributions of reduced Ni-Fe/(Mg, Al)O_x catalysts with different Ni/Fe ratios; and (e) magnified FETEM image and lattice fringes of reduced Ni/(Mg, Al)O_x catalyst.

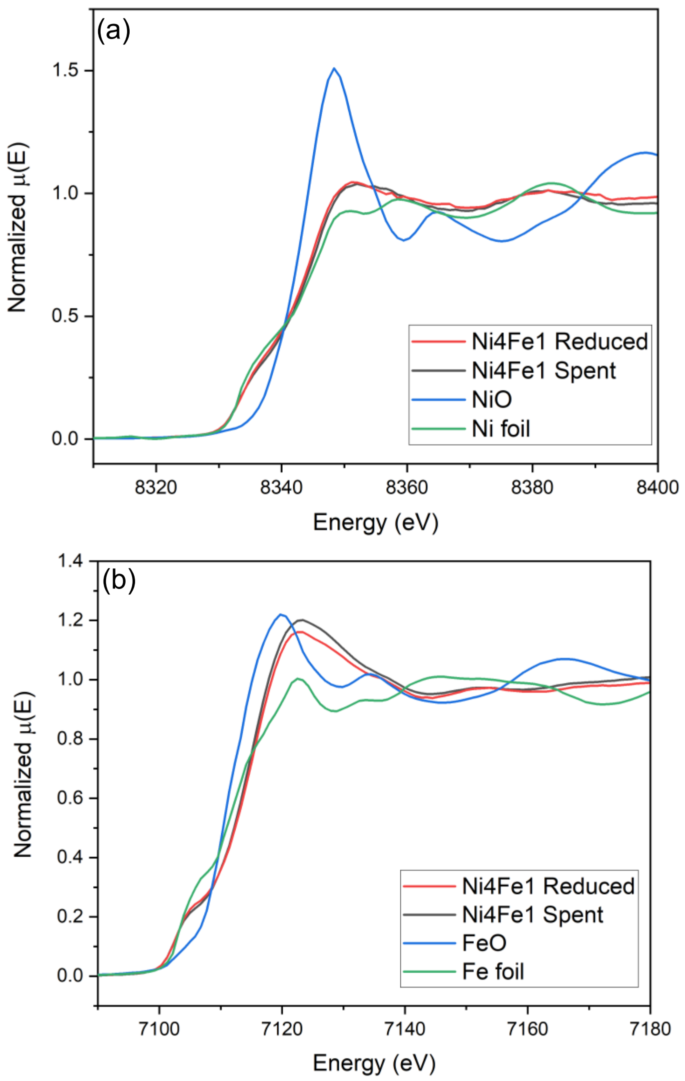

Fig. S6. (a) Ni and (b) Fe K-edge XANES spectra for reduced and spent Ni4Fe1/(Mg, Al)O_x catalysts.

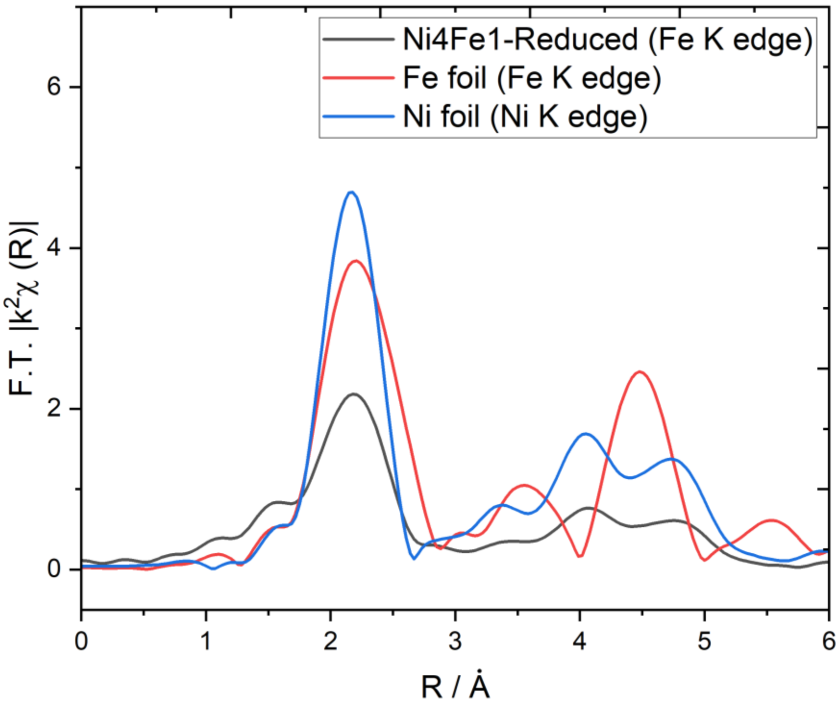

Fig. S7. Magnitude of the Fourier transform of the Fe K-edge EXAFs spectrum of the reduced Ni4Fe1/(Mg, Al)O_x catalyst, and the Ni and Fe K-edge EXAFs spectra of standard Ni and Fe foils, respectively. The Fourier transform was taken over k = 3-12.

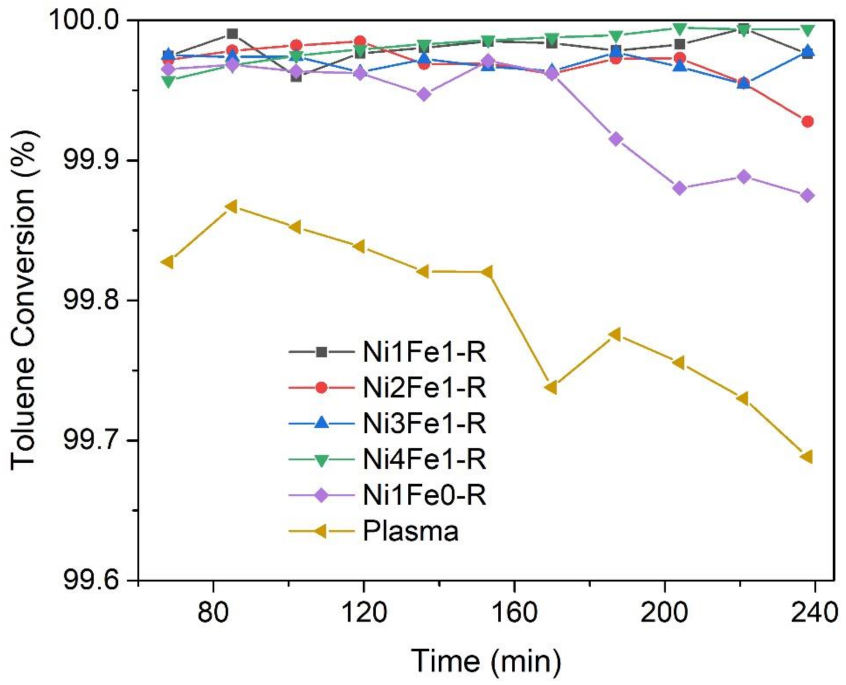

Fig. S8. Toluene conversions over reduced Ni-Fe/(Mg, Al)O_x catalysts with different Ni/Fe ratios during the plasma-catalytic CRT reaction (Reaction conditions: catalyst weight 200 mg diluted with 300 mg inert SiO_2, CO_2/toluene/Ar 7:1:63, and discharge power 36 W).

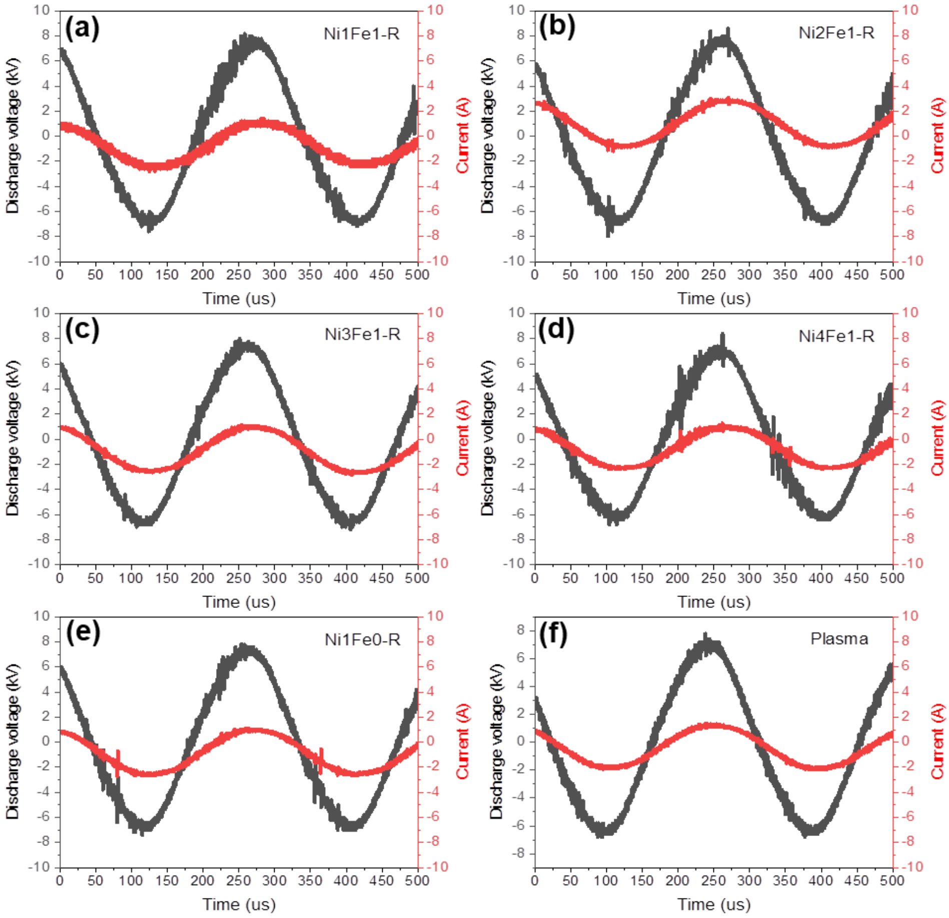

Fig. S9. Applied voltage and current signals of (a-e) DBD coupled with different Ni-Fe/(Mg, Al)O_x catalysts and (f) DBD without a catalyst.

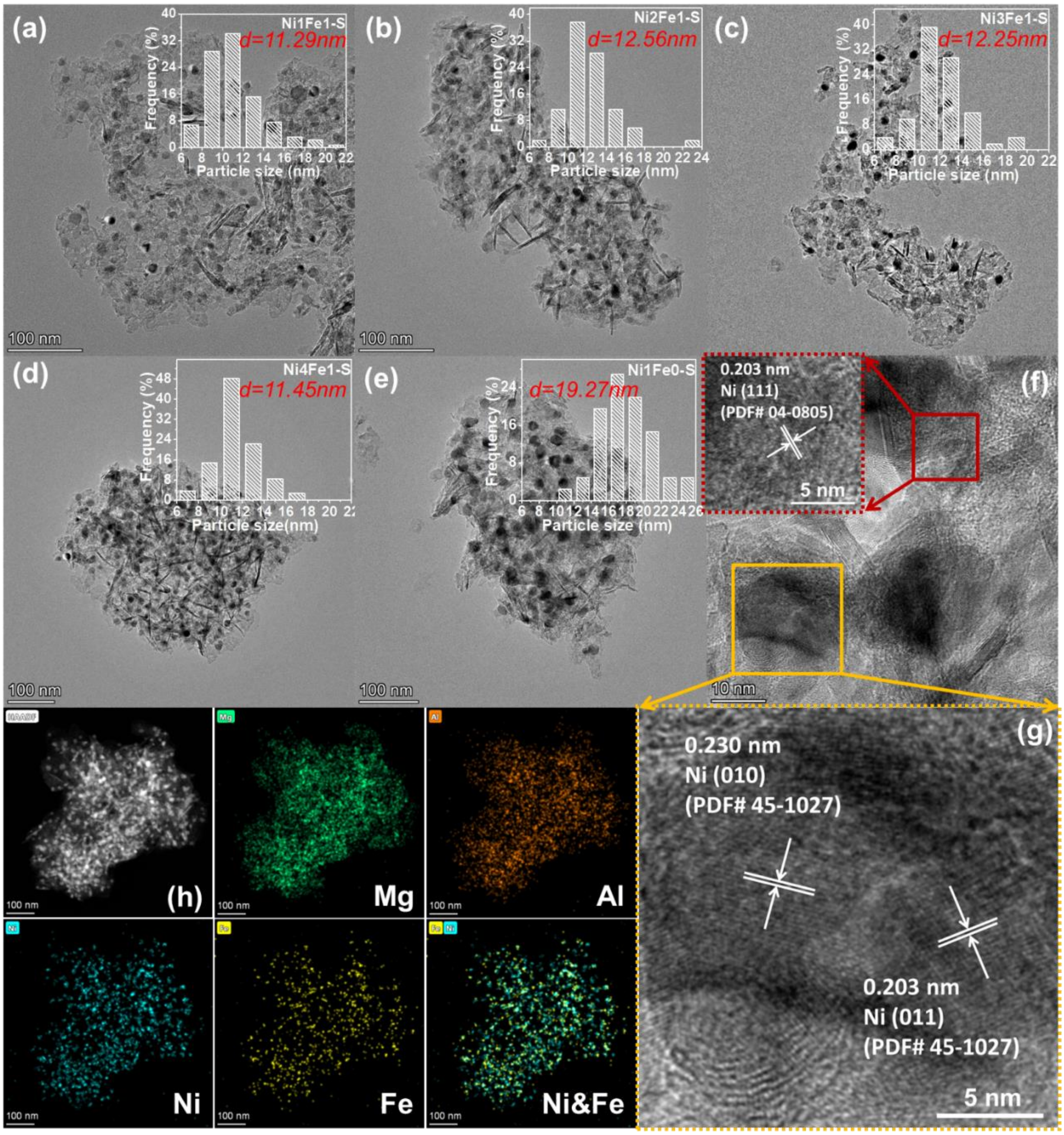

Fig. S10. FETEM images and metal particle distributions of spent (a) Ni1Fe1/(Mg, Al)O_x, (b) Ni2Fe1/(Mg, Al)O_x, (c) Ni3Fe1/(Mg, Al)O_x, (d) Ni4Fe1/(Mg, Al)O_x, and (e) Ni/(Mg, Al)O_x catalysts; (f, g) magnified FETEM images and lattice fringes of spent Ni/(Mg, Al)O_x catalysts; and (h) EDX-mapping images of spent Ni4Fe1/(Mg, Al)O_x catalyst.

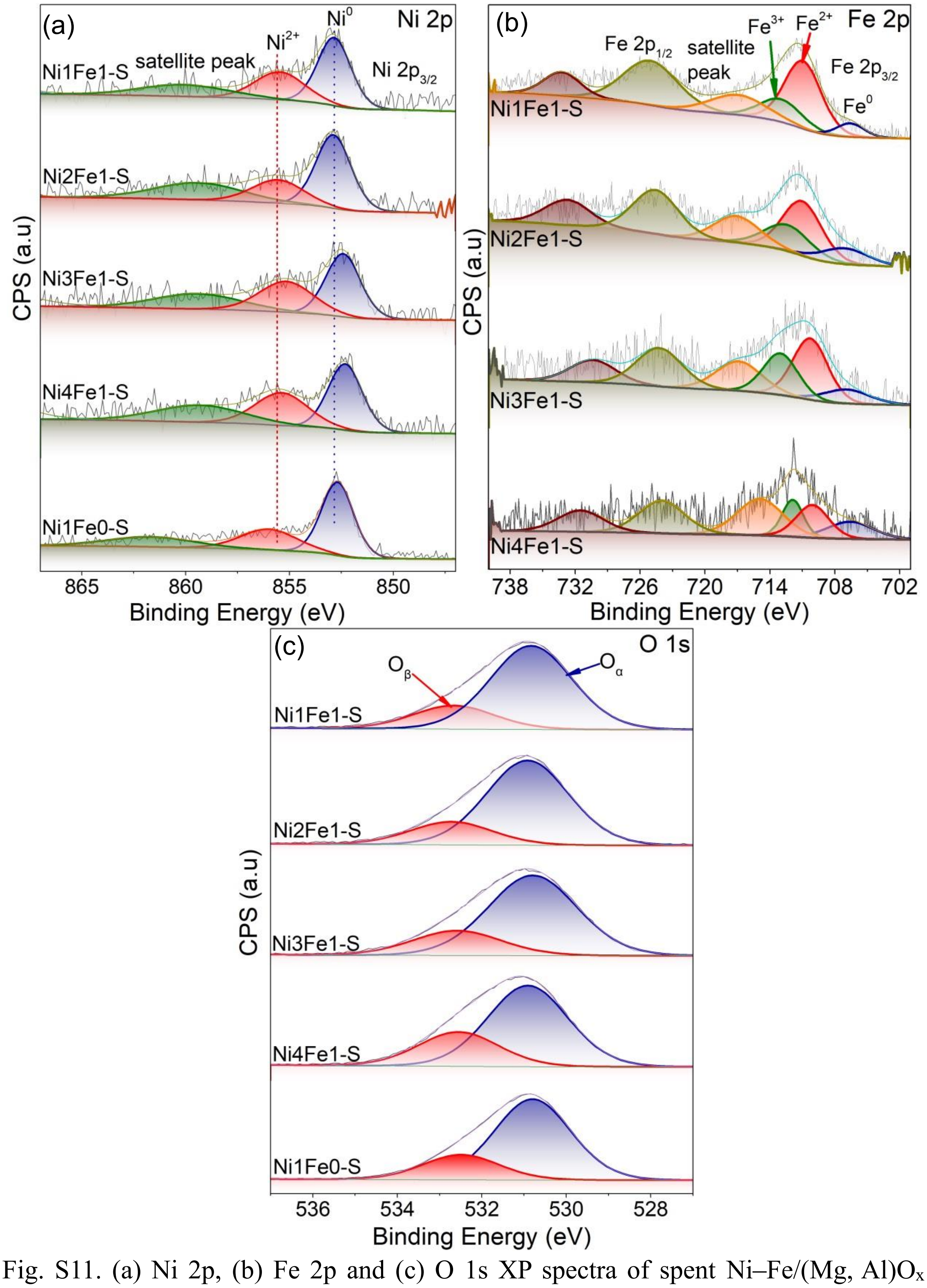

Fig. S11. (a) Ni 2p, (b) Fe 2p and (c) O 1s XP spectra of spent Ni–Fe/(Mg, Al)O_x catalysts.

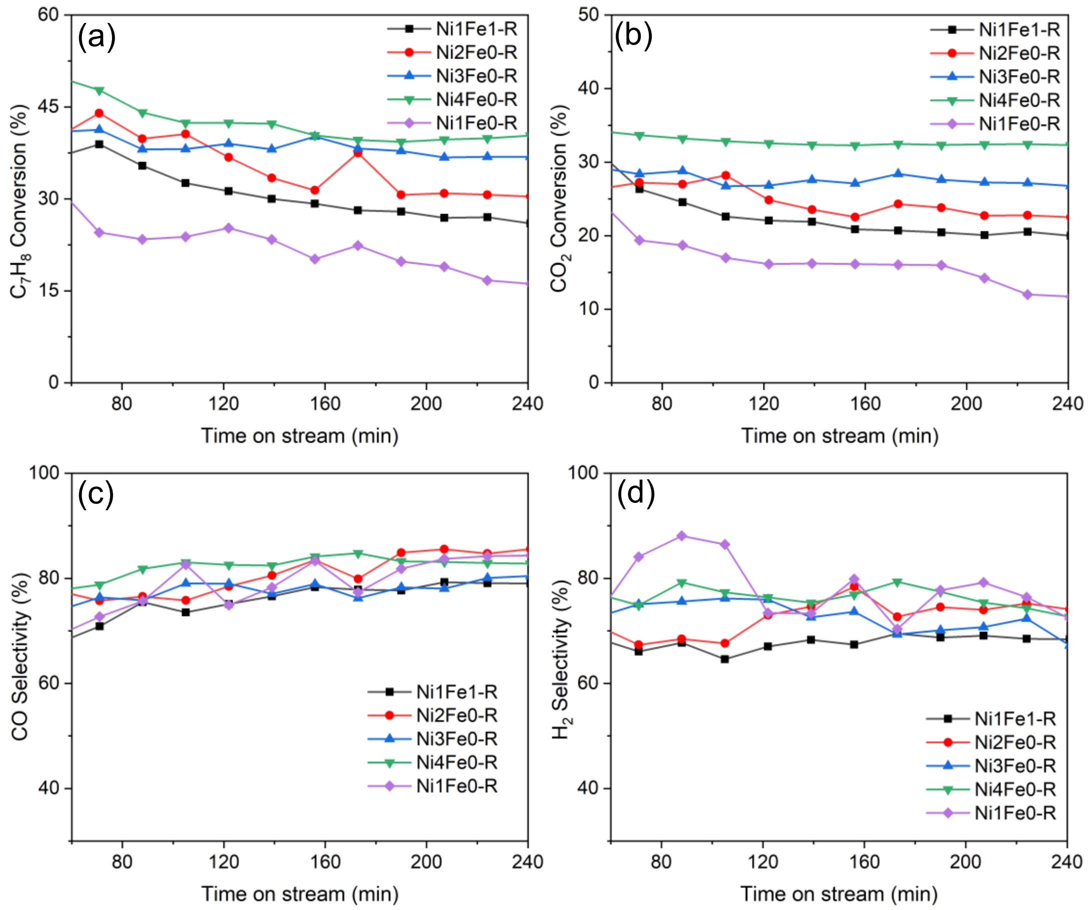

Fig. S12. Conversions of (a) toluene and (b) CO_2, and selectivity of (c) CO and (d) H_2 over reduced Ni-Fe/(Mg, Al)O_x catalysts with different Ni/Fe ratios during the thermal catalytic CRT reaction at 600 °C.

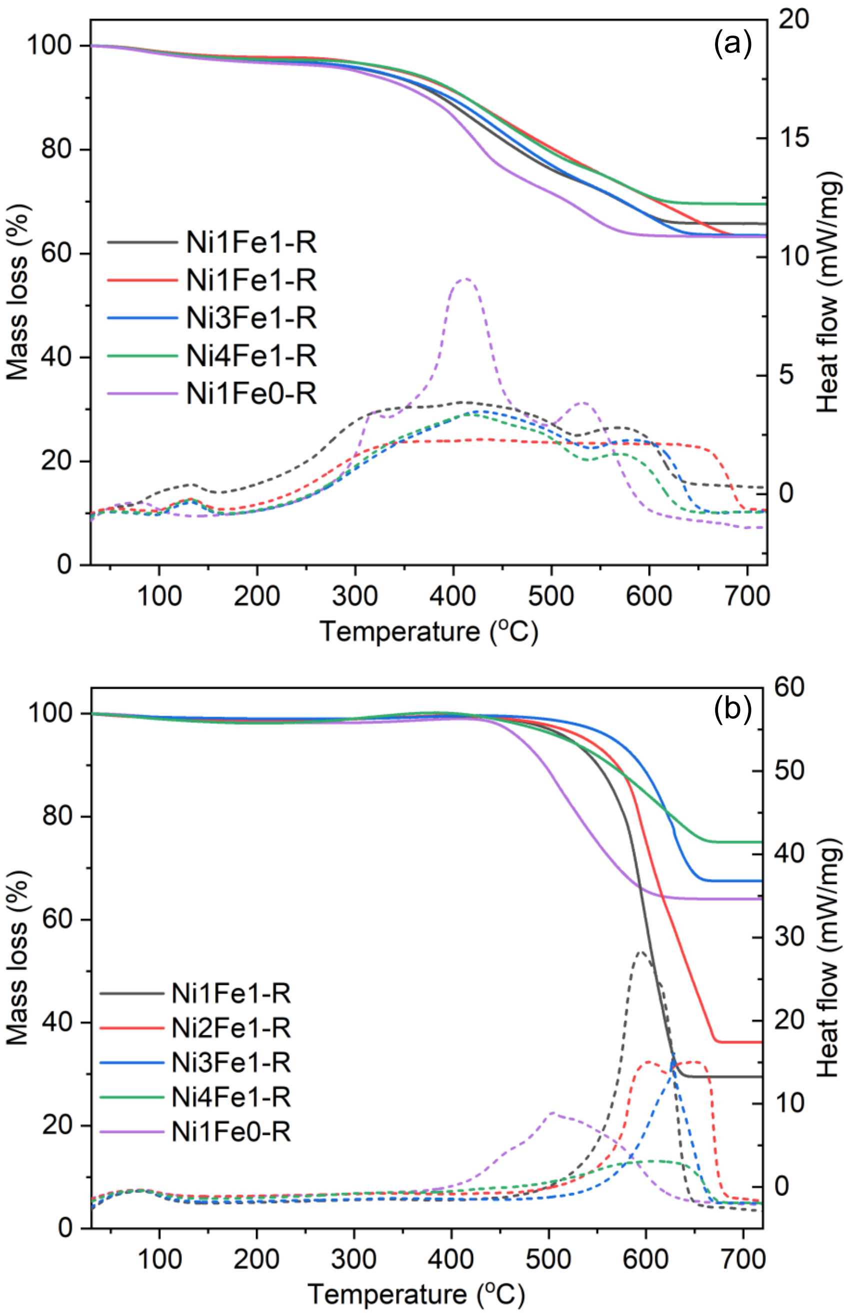

Fig. S13. TG-DSC curves of spent catalysts after (a) plasma-catalytic CRT reaction at 36 W and (b) thermal catalytic CRT reaction at 600 °C for 240 min.

Element balance of carbon, hydrogen and oxygen

The elemental balances of carbon (EB_C), hydrogen (EB_H) and oxygen (EB_O) were calculated using Eqs. (S1)-(S3), respectively, and the results are shown in **Fig. S14**. The results demonstrate that the carbon balance calculated from the production of CO, CH_4 and light hydrocarbons (C_2-C_4) was in the range of 44-52% for the plasma catalytic system, which is relatively lower than that for the thermal catalysis system due to the formation of abundant undesirable carbon-containing byproducts. As confirmed by the GC-MS and TGA results, higher hydrocarbons (e.g., benzene and p-xylene), oxygenates (maleic anhydride, benzaldehyde and benzyl alcohol) and carbon depositions are responsible for the remaining carbon output in the plasma catalytic system. H_2O is an important byproduct that contributes to the element losses of hydrogen and oxygen. However, the H_2O amount determined by hydrogen balance was higher than that determined by oxygen balance, as presented in **Table S7**. This phenomenon could be explained by the generation of abundant byproducts with high hydrogen contents, such as benzene, p-xylene, benzaldehyde and benzyl alcohol.

$$EB_C(\%) = \frac{\text{moles of carbon atoms in the products}}{7\times\text{moles of toluene converted} + \text{moles of } CO_2 \text{ converted}} \quad (S1)$$

$$EB_H(\%) = \frac{\text{moles of hydrogen atoms in the products}}{8\times\text{moles of toluene converted}} \quad (S2)$$

$$EB_O(\%) = \frac{\text{moles of oxygen atoms in CO}}{2\times\text{moles of } CO_2 \text{ converted}} \quad (S3)$$

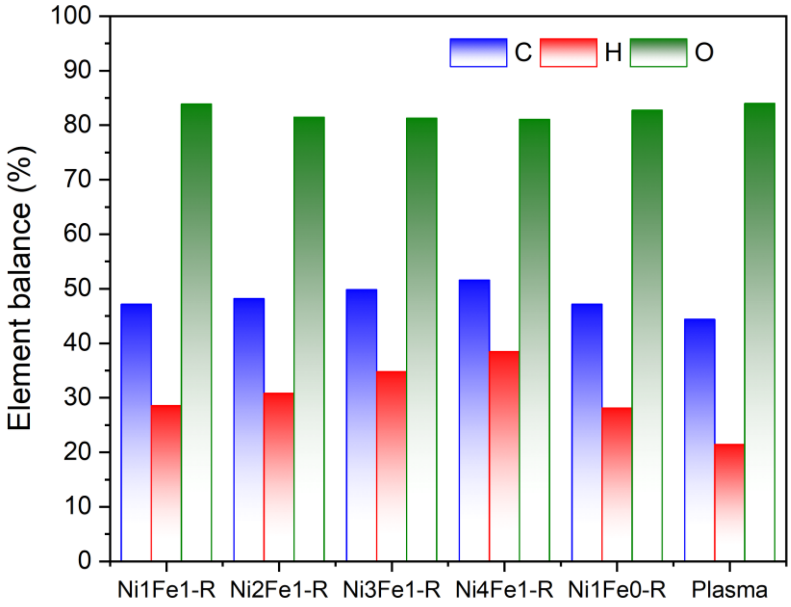

Fig. S14. Average element balance of carbon, hydrogen and oxygen during the plasma-catalytic CRT reaction (Reaction conditions: catalyst weight 200 mg diluted with 300 mg inert SiO_2, CO_2/toluene/Ar 7:1:63, discharge power 36 W and reaction time 240 min).

Interaction of CO_2 and toluene

The effect of CO_2 concentration on the performance of the CRT reaction was also examined to investigate the interaction of CO_2 and toluene, which was crucial to understanding the mechanism of the CRT reaction in the plasma-catalytic system. **Fig. S15** shows that increasing CO_2 content resulted in a continuous decrease in CO_2 conversion but an increase in CO selectivity since the conversion of CO_2 to CO and O was promoted. The direct electron impact was expected to be the most dominant pathway for the dissociation of gas phase CO_2 (Eq. (S4)), while excited Ar^* also played an important role in CO_2 dissociation (Eq. (S5)) [1]. The excited Ar^* could also excite toluene by transferring its energy in collisions to form $C_7H_8^*$ (Eq. (S6)), which was highly reactive and easily decomposed by collisions with an electron or other excited species such as O, as described in Eq. (S7). This reaction could explain the increased selectivity of H_2 as CO_2 content increased from 5 vol% to 15 vol% due to the formation of more reactive O species [2] (**Fig. S15(c)**). However, further increasing the CO_2 content to 20-30 vol.% reduced H_2 selectivity, which could be attributed to the enhanced reverse water-gas shift reaction, as shown in Eq. (S8) [3]. In this study, CH_4 might be produced by combining CH_3 and H (Eq. (S11)), which were generated by cracking toluene (Eqs. (S9) and (S10)).

To further elucidate the interaction of CO_2 and toluene, the plasma-catalytic performance of separated toluene and CO_2 dissociation was studied. The absence of CO_2 decreased H_2 selectivity significantly and the plasma discharge was quickly terminated by the spark discharge (**Fig. S15(c)**). Furthermore, the absence of toluene resulted in a much lower CO_2 conversion since the generated O species in Eq. (S4) were inefficiently consumed. However, a nearly 100% selectivity of CO was achieved due to the lack of CO-incorporated side reactions (**Fig. S16**).

$$CO_2 + e \rightarrow CO + O + e \tag{S4}$$

$$CO_2 + Ar^* \rightarrow CO + O + Ar \tag{S5}$$

$$C_7H_8 + Ar^* \rightarrow C_7H_8^* + Ar \tag{S6}$$

$$C_7H_8^* + O \rightarrow CO + H_2 + LHC \tag{S7}$$

$$CO_2 + H_2 \rightarrow CO + H_2O \tag{S8}$$

$$C_6H_5CH_3 + e \rightarrow C_6H_5CH_2 + H + e \tag{S9}$$

$$C_6H_5CH_3 + e \rightarrow C_6H_5 + CH_3 + e \tag{S10}$$

$$CH_3 + H \rightarrow CH_4 \tag{S11}$$

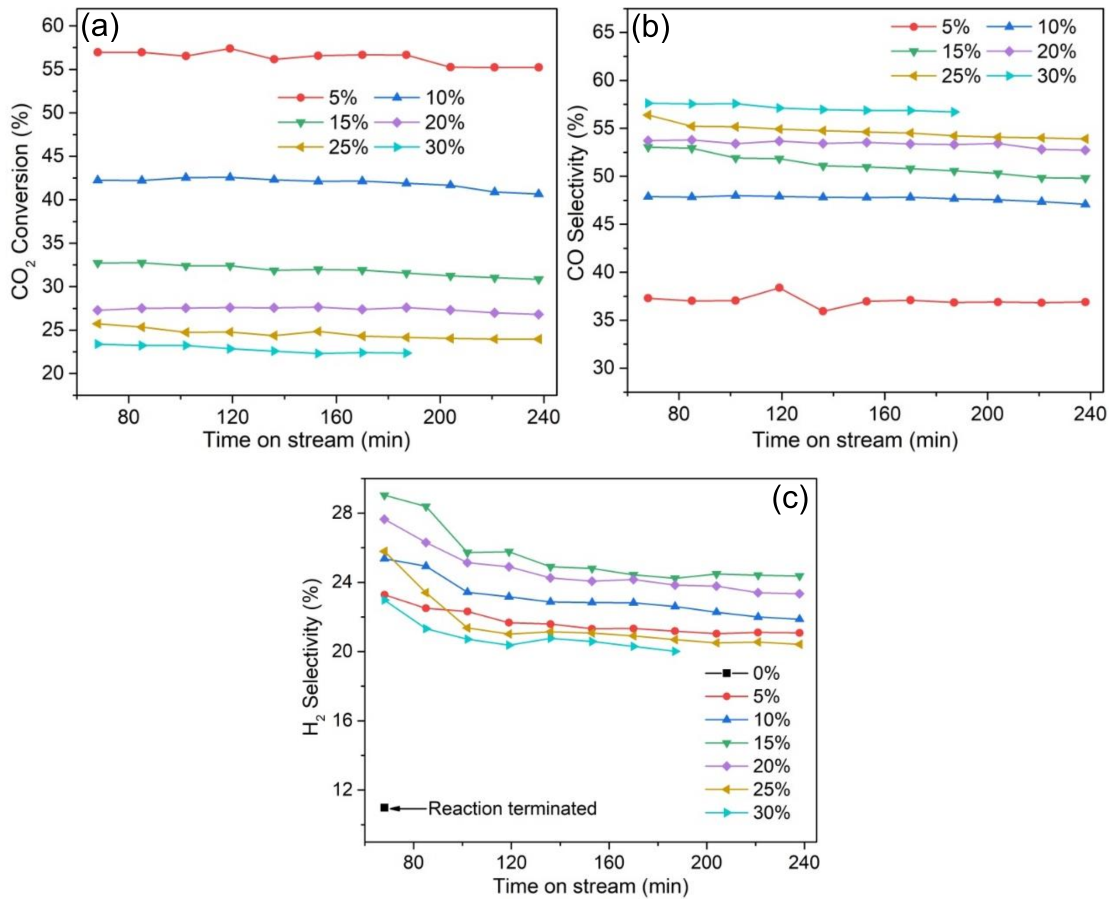

Fig. S15. Effect of CO_2 concentration on (a) conversion of CO_2, selectivity of (b) CO and (c) H_2 over the Ni4Fe1-R catalyst during the plasma-catalytic CRT reaction (Reaction condition: catalyst weight 200 mg diluted with 300 mg inert SiO_2, and discharge power 36 W).

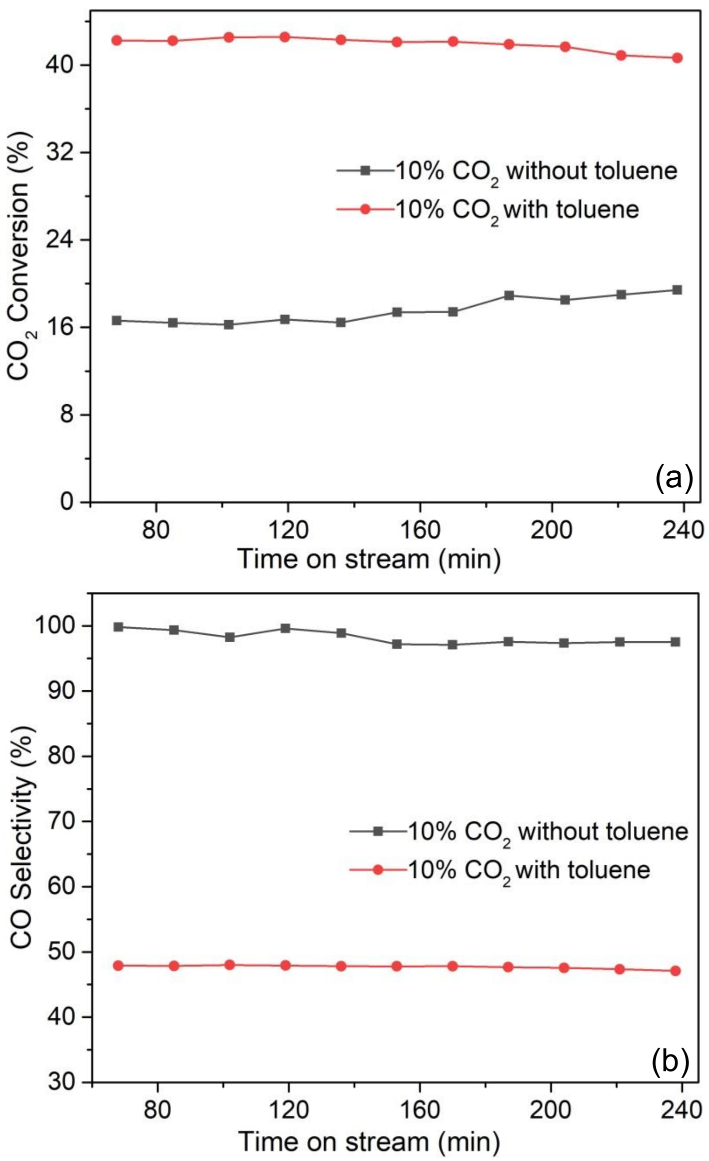

Fig. S16. (a) Conversion of CO_2 and (b) selectivity of CO in the plasma-catalytic system with and without toluene.

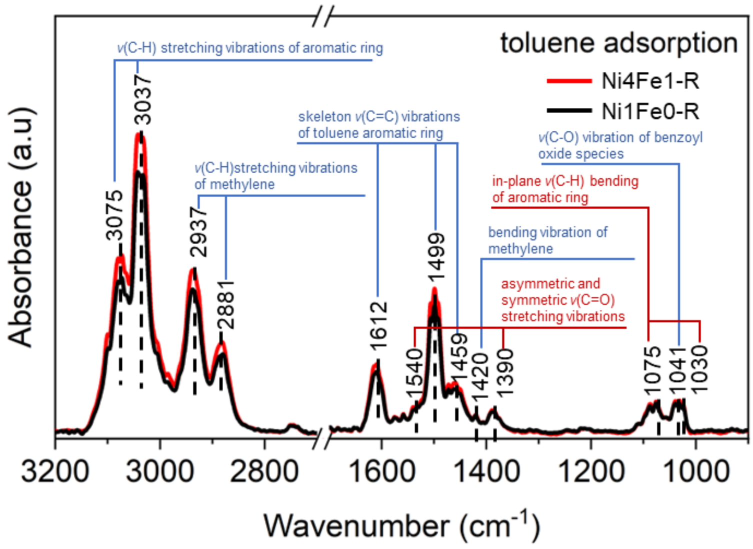

Fig. S17. FTIR spectra of toluene adsorption on the Ni4Fe1-R and Ni1Fe0-R catalysts for 30 min at 25 ºC.

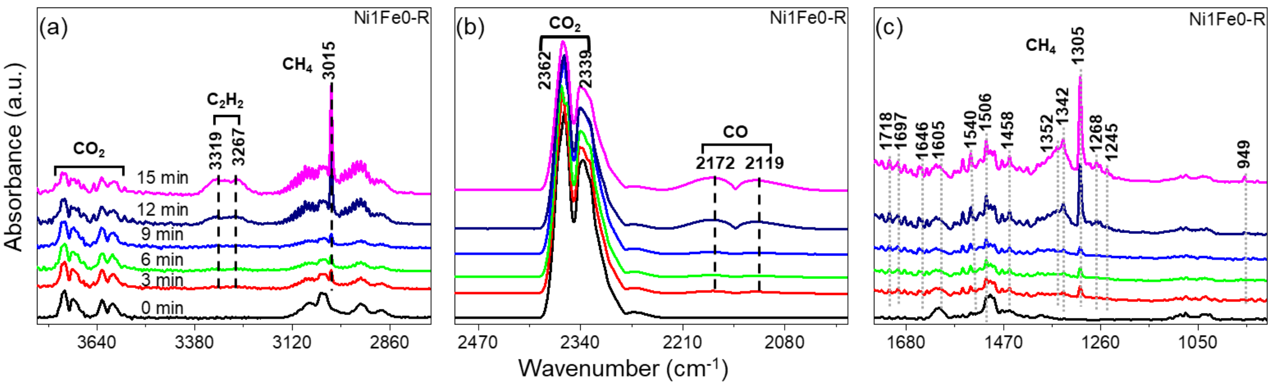

Fig. S18. *In situ* FTIR spectra of the plasma-catalytic CRT reaction over the Ni1Fe0-R catalyst at 25 °C (discharge time 15 min, feed gas 10 vol% CO_2/1.5 vol% C_7H_8 diluted in argon, total flow rate 20 ml/min).

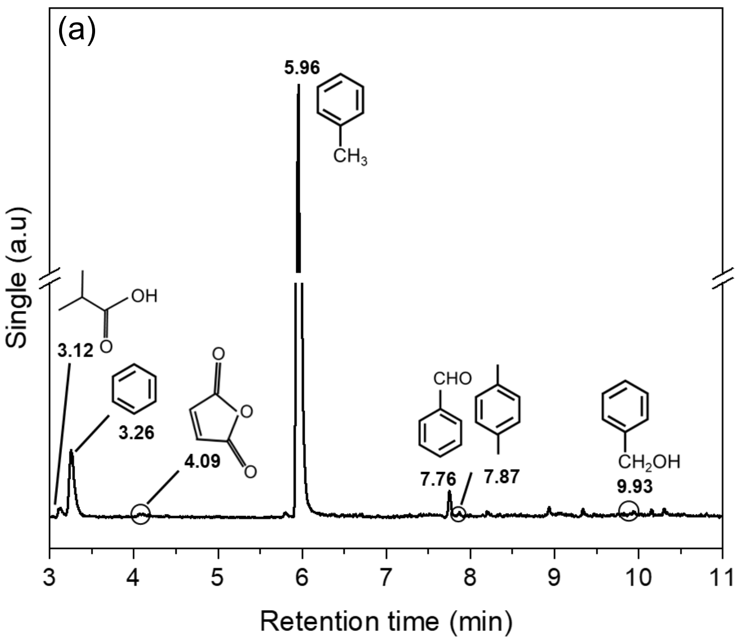

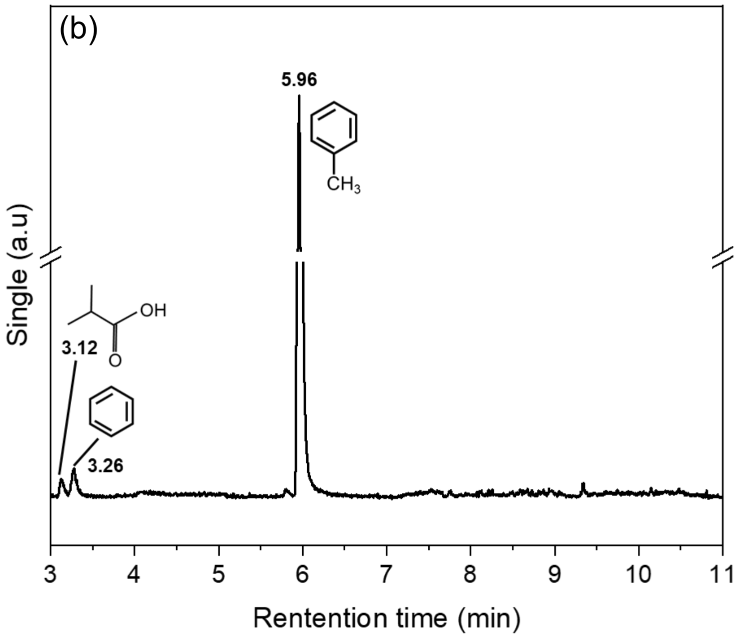

Fig. S19. GC-MS results of liquid intermediates generated in the plasma-catalytic CRT reaction over (a) Ni4Fe1-R and (b) Ni1Fe0-R catalysts.

Table S1. Chemical compositions for the preparation of hydrotalcite precursors with various Ni/Fe ratios

**Reagents**	**Ni**		**Ni4Fe1**		**Ni3Fe1**		**Ni2Fe1**		**Ni1Fe1**	
	Mole (mol)	Mass (g)	Mole (mol)	Mass (g)	Mole (mol)	Mass (g)	Mole (mol)	Mass (g)	Mole (mol)	Mass (g)
$Ni(NO_3)_2{\cdot}6H_2O$	0.024	6.98	0.019	5.58	0.018	5.23	0.016	4.65	0.012	3.49
$Fe(NO_3)_3{\cdot}9H_2O$	0	0	0.005	1.94	0.006	2.42	0.008	3.23	0.012	4.85
$Mg(NO_3)_2{\cdot}6H_2O$	0.080	20.5	0.080	20.5	0.080	20.5	0.080	20.5	0.080	20.5
$Al(NO_3)_3{\cdot}9H_2O$	0.032	12.0	0.032	12.0	0.032	12.0	0.032	12.0	0.032	12.0
M^{2+}/M^{3+} [a]	3.25		2.70		2.58		2.40		2.09	
$M^{3+}/M^{3+}+M^{2+}$ [b]	0.24		0.27		0.28		0.29		0.32	

[a] *The molar ratio of divalent metal ions (Ni^{2+} and Mg^{2+}) to trivalent metal ions (Fe^{3+} and Al^{3+});*

[b] *The value of $M^{3+}/M^{3+}+M^{2+}$ between 0.17 and 0.33 is favorable for achieving a complete hydrotalcite structure.*

Table S2. *D*-spacing and lattice parameters of the as-synthesized Ni-Fe-Mg-Al LDH precursors determined by the XRD patterns.

catalyst	*d*-spacing[a] (nm)				lattice parameters[b] (nm)	
	d(003)	*d*(006)	*d*(012)	*d*(110)	*a*	*c*
Ni1Fe1-HTs	0.7615	0.3805	0.2584	0.1528	0.3056	2.2845
Ni2Fe1-HTs	0.7660	0.3827	0.2591	0.1528	0.3056	2.2980
Ni3Fe1-HTs	0.7673	0.3841	0.2583	0.1526	0.3052	2.3019
Ni4Fe1-HTs	0.7570	0.3869	0.2592	0.1531	0.3062	2.2710
Ni1Fe0-HTs	0.7799	0.3910	0.2590	0.1528	0.3056	2.3397

[a] *Calculated according to Bragg's law;*

[b] *Calculated by the formulas: $a = 2\ d(110)$ and $c = 3d(003)$.*

Table S3. Actual and nominal element compositions of calcined catalysts determined by ICP-OES.

**Element**	**Loading (wt%)**	**Ni1Fe1-F**	**Ni2Fe1-F**	**Ni3Fe1-F**	**Ni4Fe1-F**	**Ni1Fe0-F**
Ni	Exp.[a]	10.3	13.4	14.8	15.2	19.6
	Nom.[b]	10.5	14.0	15.8	16.9	21.2
Fe	Exp.	9.2	6.7	5.1	4.1	/
	Nom.	10.0	6.7	5.0	4.0	/
Mg	Exp.	29.5	29.5	29.6	29.2	29.2
	Nom.	29.0	29.1	29.1	29.1	29.2
Al	Exp.	13.2	12.9	12.9	12.9	13.1
	Nom.	12.9	12.9	12.9	13.0	13.0
O	Exp.	37.8	37.5	37.6	38.6	38.1
Ni/Fe		1.1	2	2.9	3.7	/

[a] *Measured element composition by ICP-OES.*

[b] *Nominal element composition calculated by the stoichiometric ratio.*

Table S4. Surface element compositions of reduced and spent Ni–Fe/(Mg, Al)O_x catalysts determined by XPS spectra.

Catalyst	Surface nickel species				Surface oxygen species				Surface iron species					
	Ni^0		Ni^{2+}		Lattice oxygen		Adsorbed oxygen		Fe^0		Fe^{2+}		Fe^{3+}	
	B.E. (eV)	Area (%)	B.E. (eV)	Area (%)	B.E. (eV)	Area (%)	B.E. (eV)	Area (%)	B.E. (eV)	Area (%)	B.E. (eV)	Area (%)	B.E. (eV)	Area (%)
**Ni1Fe1-R**	852.5	66.3	855.2	33.7	530.0	41.7	531.2	58.3	706.5	19.1	709.4	22.8	711.2	58.1
**Ni2Fe1-R**	852.6	63.4	855.2	36.6	530.0	55.5	531.6	44.5	707.1	35.6	710.2	29.8	712.8	34.6
**Ni3Fe1-R**	852.0	65.3	855.8	34.7	530.2	63.2	531.9	36.8	706.8	38.4	710.0	28.8	712.2	32.8
**Ni4Fe1-R**	852.3	60.5	855.5	39.5	531.1	69.2	532.9	30.8	707.3	41.7	710.9	25.2	712.9	33.1
**Ni1Fe0-R**	852.8	59.6	856.0	40.4	531.0	62.9	532.7	37.1	/	/	/	/	/	/
**Ni1Fe1-S**	852.8	62.0	855.5	38.0	530.8	78.1	532.7	21.9	706.6	9.7	710.9	65.2	713.0	25.0
**Ni2Fe1-S**	852.9	68.2	855.5	31.2	530.9	78.6	532.7	21.4	707.0	17.8	711.0	52.6	712.4	29.6
**Ni3Fe1-S**	852.4	58.3	855.2	41.7	530.8	76.3	532.5	23.7	706.9	15.2	710.3	50.5	713.0	34.3
**Ni4Fe1-S**	852.3	57.3	855.4	42.7	530.9	70.2	532.5	29.8	706.6	29.7	710.1	40.7	711.9	29.6
**Ni1Fe0-S**	852.8	66.3	856.0	33.7	530.8	76.3	532.5	23.7	/	/	/	/	/	/

Table S5. EXAFS fitting parameters for the Ni4Fe1-R catalyst.

	Scatter	C.N.	R (Å)	σ^2 (Å^2)	E_a (eV)	R-factor
**Ni edge**	Ni-O	1.3±1.4	2.08±0.01	0.0074±0.0170	10.4±8.8	0.004
	Ni-Ni (Fe) -1st	6.1±1.4	2.49±0.01	0.0064±0.0016	6.0±2.6	
	Ni-Ni (Fe) -2nd	3.5 ±0.7	3.45±0.03	0.0098±0.0035		
**Fe edge**	Fe-O	2.8±1.5	2.08±0.06	0.0105±0.0112	5.63±4.59	0.009
	Fe-Fe (Ni)	6.9±1.4	2.52±0.01	0.0061±0.0014	-2.53±2.01	

Table S6. EXAFS fitting parameters for the Ni4Fe1-S catalyst.

	**Scatter**	**C.N.**	**R (Å)**	**σ^2 (Å^2)**	**E_a (eV)**	**R-factor**
**Ni edge**	Ni-Ni (Fe) -1st	8.4 ±0.9	2.49±0.01	0.0084±0.0010	3.7±1.2	0.011
	Ni-Ni (Fe) -2nd	4.2 ±0.5	3.47±0.02	0.0109±0.0281		
**Fe edge**	Fe-O	3.2±0.9	2.00±0.03	0.0114±0.0062	-0.95±2.93	0.005
	Fe-Fe (Ni)	5.6 ±0.8	2.52±0.01	0.0062±0.0011	-3.65±1.44	

Table S7. Estimated H_2O production rate from hydrogen and oxygen balance in the plasma-catalytic system (mmol/min).

Catalyst	Ni1Fe1-R	Ni2Fe1-R	Ni3Fe1-R	Ni4Fe1-R	Ni1Fe0-R	Plasma
**H_2O amount determined by H balance**	0.184	0.179	0.168	0.158	0.185	0.202
**H_2O amount determined by O balance**	0.056	0.067	0.069	0.074	0.060	0.049

Table S8. *In situ* FTIR bands of the surface adsorbed species on the LDH-derived Ni-Fe/(Mg, Al)O_x catalysts during the plasma-catalytic CRT reaction at 35 °C and 1 bar.

**Wavenumber/cm^{-1}**	**Assignment**	**Surface species**	**Reference**
3730, 3630	υ(O-H) vibration	bicarbonate ($^{*}HCO_3$)	[4, 5]
3700,3600	CO_2 overtones	gas-phase CO_2	[4, 6]
2362, 2339	stretching vibration of C-O bonds		
3262, 3267	vibration bands of C_2H_2	C_2H_2	[7]
3015, 1305	υ(C-H) stretching vibrations of CH_4	gas-phase methane	[6, 8]
2172, 2119	stretching vibrations of C-O bonds	gas-phase CO	[6, 9]
1718, 1506	υ(C=O) stretching vibrations of unsaturated aliphatic acid	surface maleate species	[10, 11]
1697, 1646	v(C=O) stretching vibration	benzaldehyde species	[12]
1605	asymmetric stretching vibration of COO^-	formate species	[13]
1540, 1342	asymmetric and symmetric stretching vibration of COO-	benzoate species	[12-14]
1268	C-O stretching vibration		
1458	bending vibration of C-OH	benzyl alcoholate	[12-14]
1352	CH_2 deformation vibration of benzyl species		
1245	C-O stretching vibration		
949	υ(C=C) vibration	short-chain olefin	[10]
3075,3037	υ(C-H) stretching vibration of aromatic ring	aromatic ring	[15, 16]
1612, 1499, 1459	skeleton υ(C=C) vibration of aromatic ring		
1075,1030	in-plane υ(C-H) bending of aromatic ring		
2937, 2881	symmetric or asymmetric υ(C-H) stretching of methylene	methylene (-CH_2)	[17]
1420	bending vibration of methylene		
1041	C-O vibration band	benzoyl oxide species	[18]

References

[1] K. Zhang, G. Zhang, X. Liu, A.N. Phan, K. Luo, A study on CO_2 decomposition to CO and O_2 by the combination of catalysis and dielectric-barrier discharges at low temperatures and ambient pressure, Industrial & Engineering Chemistry Research, 56 (2017) 3204-3216.

[2] K. Xiao, X. Li, J. Santoso, H. Wang, K. Zhang, J. Wu, D. Zhang, Synergistic effect of dielectric barrier discharge plasma and Mn catalyst on CO_2 reforming of toluene, Fuel, 285 (2021) 119057.

[3] F. Zhu, H. Zhang, H. Yang, J. Yan, X. Li, X. Tu, Plasma reforming of tar model compound in a rotating gliding arc reactor: Understanding the effects of CO_2 and H_2O addition, Fuel, 259 (2020) 116271.

[4] L. Proaño, E. Tello, M.A. Arellano-Trevino, S. Wang, R.J. Farrauto, M. Cobo, In-situ DRIFTS study of two-step CO_2 capture and catalytic methanation over Ru,"Na_2O"/Al_2O_3 dual functional material, Applied Surface Science, 479 (2019) 25-30.

[5] Z. Tang, W. He, Y. Wang, Y. Wei, X. Yu, J. Xiong, X. Wang, X. Zhang, Z. Zhao, J. Liu, Ternary heterojunction in rGO-coated Ag/Cu_2O catalysts for boosting selective photocatalytic CO_2 reduction into CH_4, Applied Catalysis B: Environmental, 311 (2022) 121371.

[6] C. Cerdá-Moreno, A. Chica, S. Keller, C. Rautenberg, U. Bentrup, Ni-sepiolite and Ni-todorokite as efficient CO_2 methanation catalysts: Mechanistic insight by operando DRIFTS, Applied Catalysis B: Environmental, 264 (2020) 118546.

[7] R. Vakili, R. Gholami, C.E. Stere, S. Chansai, H. Chen, S.M. Holmes, Y. Jiao, C. Hardacre, X. Fan, Plasma-assisted catalytic dry reforming of methane (DRM) over metal-organic frameworks (MOFs)-based catalysts, Applied Catalysis B: Environmental, 260 (2020) 118195.

[8] H.L. Huynh, J. Zhu, G. Zhang, Y. Shen, W.M. Tucho, Y. Ding, Z. Yu, Promoting effect of Fe on supported Ni catalysts in CO_2 methanation by in situ DRIFTS and DFT study, Journal of Catalysis, 392 (2020) 266-277.

[9] S. Das, S. Bhattar, L. Liu, Z. Wang, S. Xi, J.J. Spivey, S. Kawi, Effect of partial Fe substitution in $La_{0.9}Sr_{0.1}NiO_3$ perovskite-derived catalysts on the reaction mechanism of methane dry reforming, ACS Catalysis, 10 (2020) 12466-12486.

[10] Y. Sun, J. Han, K. Xu, K. Wu, W. Wu, X. Zhang, B. Jiang, Probing the effects of plasma-induced surface species in ring-opening process of toluene decomposition via plasma-excited TPD and in situ DRIFTS, Journal of Cleaner Production, 371 (2022) 133332.

[11] X. Zhang, F. Bi, Z. Zhu, Y. Yang, S. Zhao, J. Chen, X. Lv, Y. Wang, J. Xu, N. Liu, The promoting effect of H_2O on rod-like $MnCeO_x$ derived from MOFs for toluene oxidation: A combined experimental and theoretical investigation, Applied Catalysis B: Environmental, 297 (2021) 120393.

[12] L. Zhang, Y. Liu, X. Fang, Y. Cheng, Regulating oxygen species for promoted deep oxidation of toluene: A comparative study of adsorbed oxygen and lattice oxygen, Fuel, 321 (2022) 124116.

[13] Z. Wu, D. Zhu, Z. Chen, S. Yao, J. Li, E. Gao, W. Wang, Enhanced energy efficiency and reduced nanoparticle emission on plasma catalytic oxidation of toluene using

Au/γ-Al_2O_3 nanocatalyst, Chemical Engineering Journal, 427 (2022) 130983.
[14] J. Li, H. Na, X. Zeng, T. Zhu, Z. Liu, In situ DRIFTS investigation for the oxidation of toluene by ozone over Mn/HZSM-5, Ag/HZSM-5 and Mn–Ag/HZSM-5 catalysts, Applied Surface Science, 311 (2014) 690-696.
[15] S. Mo, Q. Zhang, J. Li, Y. Sun, Q. Ren, S. Zou, Q. Zhang, J. Lu, M. Fu, D. Mo, J. Wu, H. Huang, D. Ye, Highly efficient mesoporous MnO_2 catalysts for the total toluene oxidation: Oxygen-Vacancy defect engineering and involved intermediates using in situ DRIFTS, Applied Catalysis B: Environmental, 264 (2020) 118464.
[16] B. Jiang, K. Xu, J. Li, H. Lu, X. Fei, X. Yao, S. Yao, Z. Wu, Effect of supports on plasma catalytic decomposition of toluene using in situ plasma DRIFTS, Journal of Hazardous Materials, 405 (2021) 124203.
[17] H. Sun, Z. Liu, S. Chen, X. Quan, The role of lattice oxygen on the activity and selectivity of the OMS-2 catalyst for the total oxidation of toluene, Chemical Engineering Journal, 270 (2015) 58-65.
[18] V. Augugliaro, H. Kisch, V. Loddo, M.J. López-Muñoz, C. Márquez-Álvarez, G. Palmisano, L. Palmisano, F. Parrino, S. Yurdakal, Photocatalytic oxidation of aromatic alcohols to aldehydes in aqueous suspension of home prepared titanium dioxide: 2. Intrinsic and surface features of catalysts, Applied Catalysis A: General, 349 (2008) 189-197.
